# Spatial regularity control of phyllotaxis pattern generated by the mutual interaction between auxin and PIN1

**DOI:** 10.1371/journal.pcbi.1006065

**Published:** 2018-04-03

**Authors:** Hironori Fujita, Masayoshi Kawaguchi

**Affiliations:** 1 National Institute for Basic Biology, Okazaki, Aichi, Japan; 2 Department of Basic Biology, School of Life Science, SOKENDAI (The Graduate University for Advanced Studies), Okazaki, Aichi, Japan; Northeastern University, UNITED STATES

## Abstract

Phyllotaxis, the arrangement of leaves on a plant stem, is well known because of its beautiful geometric configuration, which is derived from the constant spacing between leaf primordia. This phyllotaxis is established by mutual interaction between a diffusible plant hormone auxin and its efflux carrier PIN1, which cooperatively generate a regular pattern of auxin maxima, small regions with high auxin concentrations, leading to leaf primordia. However, the molecular mechanism of the regular pattern of auxin maxima is still largely unknown. To better understand how the phyllotaxis pattern is controlled, we investigated mathematical models based on the auxin–PIN1 interaction through linear stability analysis and numerical simulations, focusing on the spatial regularity control of auxin maxima. As in previous reports, we first confirmed that this spatial regularity can be reproduced by a highly simplified and abstract model. However, this model lacks the extracellular region and is not appropriate for considering the molecular mechanism. Thus, we investigated how auxin maxima patterns are affected under more realistic conditions. We found that the spatial regularity is eliminated by introducing the extracellular region, even in the presence of direct diffusion between cells or between extracellular spaces, and this strongly suggests the existence of an unknown molecular mechanism. To unravel this mechanism, we assumed a diffusible molecule to verify various feedback interactions with auxin–PIN1 dynamics. We revealed that regular patterns can be restored by a diffusible molecule that mediates the signaling from auxin to PIN1 polarization. Furthermore, as in the one-dimensional case, similar results are observed in the two-dimensional space. These results provide a great insight into the theoretical and molecular basis for understanding the phyllotaxis pattern. Our theoretical analysis strongly predicts a diffusible molecule that is pivotal for the phyllotaxis pattern but is yet to be determined experimentally.

## Author summary

Self-organization of spatially regular patterns is critical for development and differentiation in multicellular organisms. Phyllotaxis, the arrangement of leaves on a plant stem, shows diverse patterns depending on plant species, which attracts many people because of its beautiful geometric configuration. In particular, it is well known that the spiral phyllotaxis is closely related to mathematical concepts such as the golden ratio and Fibonacci sequence. The phyllotaxis pattern is established by the mutual interaction between a diffusible plant hormone auxin and its efflux carrier PIN1, but its molecular mechanism is still largely unknown. To understand how phyllotaxis the pattern is controlled, we have theoretically and numerically investigated mathematical models based on the auxin–PIN1 interaction. Our theoretical analysis predicts a diffusible molecule that is critical for the phyllotaxis pattern but is yet to be determined experimentally. Furthermore, we predict the molecular mechanism of this molecule, which must mediate the feedback signaling from auxin to PIN1 polarization. Our study provides a detailed insight into the theoretical and molecular basis for understanding the phyllotaxis pattern, leading to the creation of plant varieties with various phyllotaxis patterns in the future.

## Introduction

Living organisms often form periodic patterns with spatial regularity in a self-organizing manner [1, 2]. One such well-known example is phyllotaxis, the arrangement of leaves on a plant stem. The phyllotaxis exhibits various types of patterns depending on the plant species and this has attracted many scientists because of its beautiful geometric configuration [3]. Phyllotaxis is originated at the shoot meristem, in which leaf primordia are periodically formed by maintaining a constant distance from each other [4–7]. This spatial regularity is established by the mutual interaction between a mobile plant hormone auxin and its efflux carrier membrane protein PIN1, which cooperatively generate small regions with high auxin concentrations called auxin maxima that are involved in leaf primordia. In the process of the auxin maxima formation, auxin accumulates at the presumptive position of a future primordium while PIN1 is polarized toward the center position [8–11]. According to this experimental observation, the auxin maxima pattern is often explained by the concept of “up-the-gradient” in which auxin is transported by PIN1 against its own gradient while PIN1 is polarized toward higher auxin [11–13].

Based on this concept, a class of mathematical models (corresponding to Model O in this paper) has been proposed, in which PIN1 is localized to a cell membrane depending on the auxin concentration of neighboring cells. Because these models can successfully reproduce the spatial regularity of auxin maxima and various phyllotaxis types, they are excellent models for understanding the nature of phyllotaxis pattern formation [11, 12, 14–16]. On the other hand, they are highly simplified and abstract models, and could have various problems when considering the molecular mechanism. With respect to spatial structure, these can be distilled into two major points. First, extracellular space (i.e., apoplast space) is absent in these models, in which auxin moves directly between cells by PIN1 and diffusion. However, in plant tissues, auxin is transported between cytoplasm and apoplast because cells do not contact each other but are separated by apoplast space. Second, it remains unclear how cells sense auxin concentrations of neighboring but separated cells for PIN1 polarization.

Sahlin *et al*. [16] showed that, despite the extracellular space, self-organized patterns can be generated by the “up-the-gradient” concept that PIN1 polarization depends on the auxin concentration of neighboring cells. However, it has still not been clarified how the information on auxin concentration is transmitted between neighboring cells. Conversely, although the apoplast space is also considered by Webnik *et al*. [17] and Cieslak *et al*. [18], these reports describe the canalization pattern generated by the “with-the-flux” concept, which is different from auxin maxima by the “up-the-gradient” concept.

In this paper, therefore, we investigated how the spatial regularity of phyllotaxis pattern is controlled under realistic conditions, that is, in the presence of extracellular space. We first confirmed that the introduction of extracellular space has a disruptive effect on the spatial regularity in the conventional model (Model O), even in the presence of direct auxin diffusion between cytoplasm or apoplast spaces. This result strongly suggests that an unknown molecular mechanism is required for phyllotaxis pattern formation. We also found that the spatial regularity can be restored by assuming a diffusible molecule that mediates the feedback signaling from auxin to PIN1 polarization. This theoretical analysis strongly predicts a diffusible molecule that is critical for phyllotaxis pattern but remains to be found.

## Models

**Fig 1:**
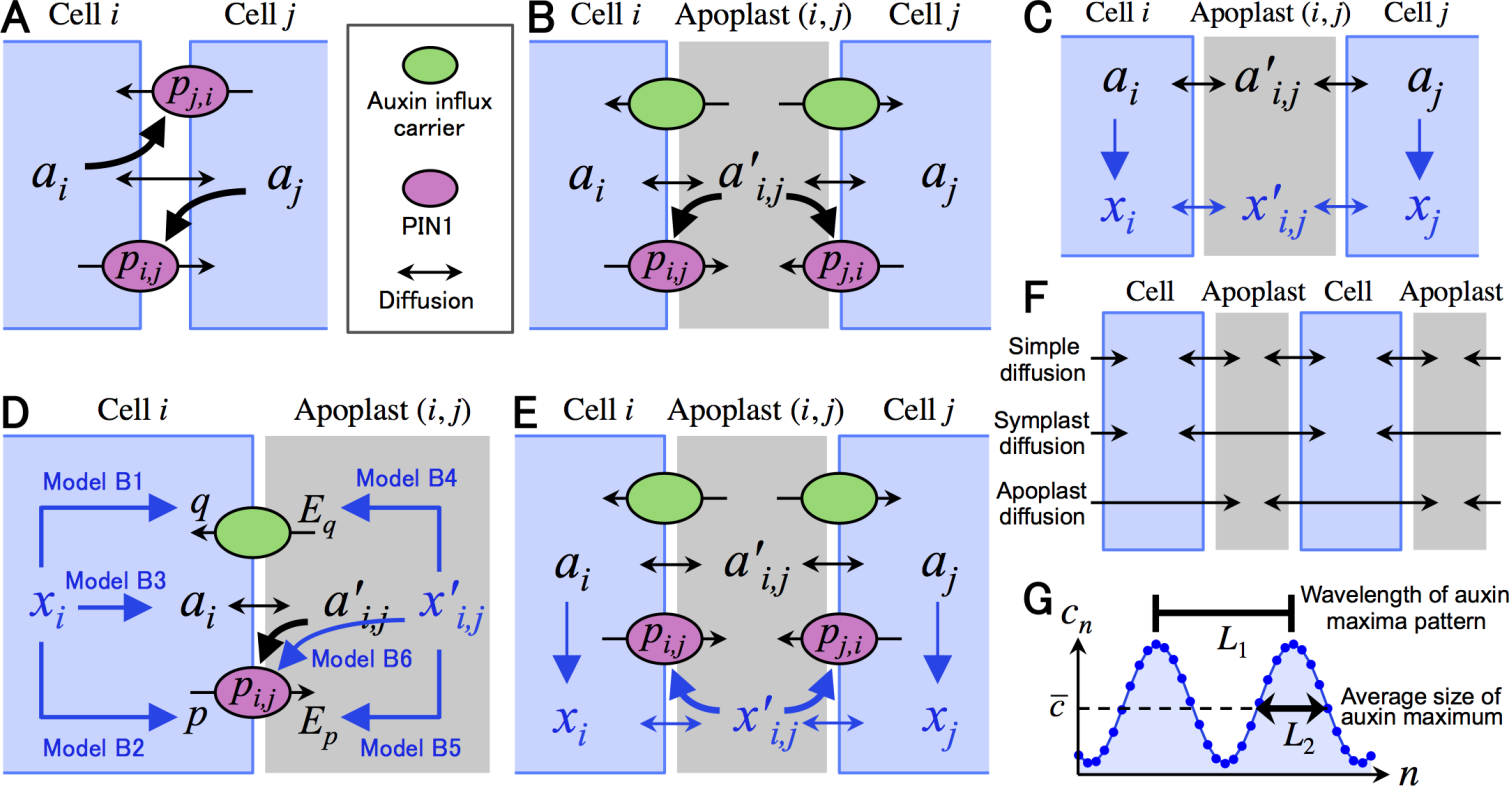
Schematic representations of models. (A) In Model O, auxin is transported between neighboring cells by PIN1, while PIN1 is polarized depending on auxin concentration of neighboring cells. (B) In Model A, auxin is transported between cytoplasm and apoplast by PIN1 and influx carrier, while PIN1 is polarized depending on auxin concentration of neighboring apoplast spaces. (C) In the framework of Model B, an assumed molecule *X* is incorporated into Model A. Molecule *X* is expressed in response to cytosolic auxin and diffuses freely between cytoplasm and apoplast. (D) Model B considers various feedback regulations from molecule *X* to auxin–PIN1 dynamics. (E) In Model B6, PIN1 is polarized depending on *X* concentration of neighboring apoplast spaces, instead of auxin. (F) In addition to simple diffusion between cytoplasm and apoplast, Models A and B consider direct diffusions between neighboring cells (symplast diffusion) and between neighboring apoplast spaces (apoplast diffusion). (G) Schematic representation of *L*_1_ and *L*_2_: indices for spatial scale of auxin maxima pattern in numerical simulations. *a*_*i*_ (or *x*_*i*_) and $a_{i,j}^{\prime }$ (or $x_{i,j}^{\prime }$) are auxin (or *X*) concentrations in cell *i* and apoplast (*i*, *j*), respectively. *p*_*i*,*j*_ is PIN1 density of the membrane toward cell *j* in cell *i*.

Cells are tightly arranged in a one- or two-dimensional space. Auxin (Models O, A, and B) and a hypothesized molecule *X* (Model B) are uniformly distributed in a cell, and their concentrations in cell *i* are denoted by *a*_*i*_ and *x*_*i*_, respectively (Fig 1). Auxin efflux carrier PIN1 is unevenly distributed to the cell membrane, and its density in the membrane of cell *i* toward neighboring cell *j* is denoted by *p*_*i*,*j*_. Models A and B consider apoplast (*i*, *j*), the extracellular space between neighboring cells *i* and *j*, and concentrations of auxin and molecule *X* in apoplast (*i*, *j*) are denoted by $a_{i,j}^{\prime }(=a_{j,i}^{\prime })$ and $x_{i,j}^{\prime }(=x_{j,i}^{\prime })$, respectively.

### Model O

Change of auxin concentration of cell *i* (*a*_*i*_) is described by

$$\frac{da_{i}}{dt}=G_{a}(A-a_{i})-\sum_{j}f_{i,j}+\sum_{j}D_{a}(a_{j}-a_{i})$$ (1)

$$f_{i,j}=E_{p}(p_{i,j}a_{i}-p_{j,i}a_{j})$$ (2)

where cell *j* is a neighbor of cell *i*, *A* is related to the synthesis rate, *G*_*a*_ is the degradation rate, *D*_*a*_ is the diffusion coefficient, *E*_*p*_ is the efficiency of PIN1 efflux carrier, and *f*_*i*,*j*_(= −*f*_*j*,*i*_) is the net flow of auxin by PIN1 from cell *i* to cell *j*, consisting of auxin efflux and influx. Auxin is constantly synthesized and degraded at a constant rate (the first term of the right-hand side of Eq 1), is transported by PIN1 (the second term), and diffuses between neighboring cells (the third term). On the other hand, change of PIN1 density (*p*_*i*,*j*_) is described by

$$\frac{dp_{i,j}}{dt}=G_{p}\left(Kp\frac{\varphi_{0}(a_{j})}{\sum_{j}\varphi_{0}(a_{j})}-p_{i,j}\right)$$ (3)

where *G*_*p*_ is the degradation rate, *K* is the number of neighboring cells, *p* is a constant related to PIN1 density, and *φ*_0_(*a*_*j*_) is the regulatory function for PIN1 polarization (Fig 1A). PIN1 is localized to cell membrane depending on the auxin concentration of neighboring cells and is degraded at a constant rate. The total PIN1 amount of cell *i*, *P*_*i*_ ≡ ∑_*j*_*p*_*i*,*j*_, satisfies *dP*_*i*_/*dt* = *G*_*p*_(*Kp* − *P*_*i*_), indicating that *Kp* is the stable equilibrium of *P*_*i*_. Thus, equilibria of *a*_*i*_ and *p*_*i*,*j*_ are given respectively by

$$a_{eq}=A\;\mathrm{and}\;p_{eq}=p$$ (4)

When *G*_*p*_ is sufficiently large, *p*_*i*,*j*_ quickly approaches equilibrium:

$$p_{i,j}=Kp\frac{\varphi_{0}(a_{j})}{\sum_{j}\varphi_{0}(a_{j})}$$ (5)

Therefore, Eq 5 can be used instead of Eq 3 in a simplified version of Model O. Model O in this study is equivalent to models reported previously in references [11] and [12]. Model equations of the simplest form in [11] can be described by

$$\frac{da_{i}}{dt}=-\sum_{j}T(p_{i,j}a_{i}-p_{j,i}a_{j})+\sum_{j}D(a_{j}-a_{i})$$ (6)

$$p_{i,j}=P\frac{a_{j}}{\sum_{j}a_{j}}$$ (7)

where *T*, *D*, and *P* are constants. Eqs 6 and 7 are identical to the simplified version of Model O (Eqs 1, 2 and 5) with *G*_*a*_ = 0, *D*_*a*_ = *D*, *E*_*p*_ = *T*, *p* = *P/K*, and *φ*_0_(*a*_*j*_) = *a*_*j*_.

Conversely, equations used in [12] are somewhat complicated compared to those in [11]. However, phyllotactic patterns can be generated under the condition of fixed total PIN1 concentration (i.e., [*PIN*]_*i*_ is constant in Eq 2 of [12]), no saturation of auxin synthesis (i.e., *κ*_*IAA*_ = 0 in Eq 5), and the linear dependence of the flux on auxin concentration (i.e., replacement of ${\left[PIN\right]}_{i}^{2}$ and ${\left[PIN\right]}_{j}^{2}$ by [*PIN*]_*i*_ and [*PIN*]_*j*_, respectively, in Eq 3). In addition to these conditions, by considering a regular cell lattice (i.e., cell side length *l*_*i*→*j*_ is constant in Eq 2), model equations can be simplified by

$$\frac{da_{i}}{dt}=\rho_{IAA}-\mu_{IAA}a_{i}-\sum_{j}f_{i,j}+\sum_{j}D(a_{j}-a_{i})$$ (8)

$$f_{i,j}=T\left(p_{i,j}\frac{a_{i}}{1+\kappa_{T}a_{j}}-p_{j,i}\frac{a_{j}}{1+\kappa_{T}a_{i}}\right)$$ (9)

$$p_{i,j}=P\frac{b^{a_{j}}}{\sum_{j}b^{a_{j}}}$$ (10)

where *ρ*_*IAA*_, *μ*_*IAA*_, *D*, *T*, *κ*_*T*_, *P*, and *b* are constants. Eqs 8–10 are identical to the simplified Model O (Eqs 1, 2 and 5) with *G*_*a*_ = *μ*_*IAA*_, *A* = *ρ*_*IAA*_/*μ*_*IAA*_, *D*_*a*_ = *D*, *E*_*p*_ = *T*, *p* = *P*/*K*, and $\varphi_{0}(a_{j})=b^{a_{j}}$, except for the saturation effect of auxin from neighboring cells on the flux in Eq 9. This effect would negatively affect the pattern formation in a manner that the saturation effect becomes strong and accordingly the pattern tends to disappear as *κ*_*T*_ increases. Therefore, this effect is not essential for generating a phyllotactic pattern, indicating that Eqs 8–10 are equivalent to Model O.

### Model A

#### Incorporation of extracellular region

Model A was constructed by incorporating apoplast (*i*, *j*), the extracellular space between neighboring cells *i* and *j*, into Model O (Fig 1B). Auxin concentration of apoplast (*i*, *j*) is denoted by $a_{i,j}^{\prime }(=a_{j,i}^{\prime })$. Changes of cytosolic auxin (*a*_*i*_) and apoplast auxin ($a_{i,j}^{\prime }$) are described by

$$\frac{da_{i}}{dt}=G_{a}(A-a_{i})-\sum_{j}f_{i,j}+\sum_{j}D_{a}(a_{i,j}^{\prime }-a_{i})$$ (11)

$$\frac{da_{i,j}^{\prime }}{dt}=-G_{a}a_{i,j}^{\prime }+\frac{1}{V}(f_{i,j}+f_{j,i})+\frac{D_{a}}{V}(a_{i}+a_{j}-2a_{i,j}^{\prime })$$ (12)

$$f_{i,j}=E_{p}p_{i,j}a_{i}-E_{q}qa_{i,j}^{\prime }$$ (13)

where *G*_*a*_, *A*, and *E*_*p*_ have the same notations as in Eqs 1 and 2, *D*_*a*_ is the diffusion coefficient between cytoplasm and apoplast, *V* is the volume ratio of apoplast to cytoplasm, *E*_*q*_ is the efficiency of influx carrier function, *q* is influx carrier density of a cell side, and *f*_*i*,*j*_ is the auxin flow from cell *i* to apoplast (*i*, *j*), consisting of efflux by PIN1 and influx by auxin influx carrier. Auxin is synthesized constantly in cytoplasm and degraded at a constant rate (the first terms of the right-hand sides of Eqs 11 and 12), is transported by carriers (the second terms), and diffuses between cytoplasm and apoplast (the third terms). On the other hand, the change of PIN1 density (*p*_*i*,*j*_) is described by

$$\frac{dp_{i,j}}{dt}=G_{p}\left(Kp\frac{\varphi_{a}(a_{i,j}^{\prime })}{\sum_{j}\varphi_{a}(a_{i,j}^{\prime })}-p_{i,j}\right)$$ (14)

where *G*_*p*_, *K*, and *p* have the same notations as in Eq 3, and $\varphi_{a}(a_{i,j}^{\prime })$ is the regulatory function for PIN1 polarization. PIN1 is localized to cell membrane depending on the auxin concentration of neighboring apoplast spaces and is degraded at a constant rate (Fig 1B). As in Model O, *Kp* is the stable equilibrium of the total PIN1 amount of a cell. Equilibria of *a*_*i*_, $a_{i,j}^{\prime }$, and *p*_*i*,*j*_ are given respectively by

$$a_{eq}=(2(E_{q}q+D_{a})+VG_{a})a_{0},\;a_{eq}^{\prime }=2(E_{p}p+D_{a})a_{0},\;\mathrm{and}\;p_{eq}=p$$ (15)

where *a*_0_ ≡ *A*/(*KV*(*E*_*p*_*p* + *D*_*a*_) + 2(*E*_*q*_*q* + *D*_*a*_) + *VG*_*a*_).

#### Effect of symplast or apoplast diffusion of auxin

In addition to simple diffusion between cytoplasm and apoplast as described above, signal molecules in plants have two major diffusion types (Fig 1F). One is symplast diffusion, which is the direct diffusion between cells via narrow tube-like structures called plasmodesmata, through which small molecules including small RNAs and transcription factors can migrate between neighboring cells [19–21]. Auxin is a small signal molecule and is reported to pass through plasmodesmata [22–24]. The other type is apoplast diffusion by which signal molecules such as secreted peptides can freely move among extracellular spaces because they are connected to each other in plant tissues [25, 26]. Thus, we investigated the effect of the symplast or apoplast diffusion of auxin on pattern formation.

In Model A, the symplast diffusion of auxin is incorporated by replacing Eq 11 with

$$\frac{da_{i}}{dt}=G_{a}(A-a_{i})-\sum_{j}f_{i,j}+\sum_{j}D_{a}(a_{i,j}^{\prime }-a_{i})+\sum_{j}D_{a1}(a_{j}-a_{i})$$ (16)

where *D*_*a*1_ is the diffusion coefficient between neighboring cells. On the other hand, the apoplast diffusion of auxin is incorporated by replacing Eq 12 with

$$\frac{da_{i,j}^{\prime }}{dt}=-G_{a}a_{i,j}^{\prime }+\frac{1}{V}(f_{i,j}+f_{j,i})+\frac{D_{a}}{V}(a_{i}+a_{j}-2a_{i,j}^{\prime })+\sum_{\left(k,l\right)}\frac{D_{a2}}{V}(a_{k,l}^{\prime }-a_{i,j}^{\prime })$$ (17)

where apoplast (*k*, *l*) is a neighbor of apoplast (*i*, *j*), and *D*_*a*2_ is the diffusion coefficient between neighboring apoplast spaces.

### Model B

#### Incorporation of diffusible molecule

Model B was constructed by incorporating an assumed diffusible molecule *X* into Model A (Fig 1C). As with auxin, concentrations of molecule *X* in cell *i* and apoplast (*i*, *j*) are denoted by *x*_*i*_ and $x_{i,j}^{\prime }(=x_{j,i}^{\prime })$, respectively. Changes of *x*_*i*_ and $x_{i,j}^{\prime }$ are described respectively by

$$\frac{dx_{i}}{dt}=G_{x}(\theta (a_{i})-x_{i})+\sum_{j}D_{x}(x_{i,j}^{\prime }-x_{i})$$ (18)

$$\frac{dx_{i,j}^{\prime }}{dt}=-G_{x}x_{i,j}^{\prime }+\frac{D_{x}}{V}(x_{i}+x_{j}-2x_{i,j}^{\prime })$$ (19)

where *G*_*x*_ is the degradation rate, *D*_*x*_ is the diffusion coefficient between cytoplasm, *V* is the volume ratio of apoplast to cytoplasm, and *θ*(*a*_*i*_) is the regulatory function of auxin on *X* synthesis. Molecule *X* is synthesized depending on cytosolic auxin and degraded at a constant rate (the first terms of the right-hand sides of Eqs 18 and 19) and diffuses between cytoplasm and apoplast (the second terms) (Fig 1C). We used Eqs 11–14, 18 and 19 as the framework of Model B. Equilibria of *x*_*i*_ and $x_{i,j}^{\prime }$ are given respectively by

$$x_{eq}=(2D_{x}+VG_{x})x_{0}\;\mathrm{and}\;x_{eq}^{\prime }=2D_{x}x_{0}$$ (20)

where *x*_0_ ≡ *θ*(*a*_*eq*_)/((*KV* + 2)*D*_*x*_ + *VG*_*x*_).

#### Feedback regulations by diffusible molecule *X*

We incorporated various feedback regulations from molecule *X* to auxin-PIN1 dynamics into the Model B framework (Fig 1D; Eqs 11–14, 18 and 19) by replacements as follows:

(Model B1) Effect of cytosolic *X* (*x*_*i*_) on influx carrier amount (*q*) is incorporated by the replacement of *q* → *ψ*_1_(*x*_*i*_)*q* in Eq 13:

$$f_{i,j}=E_{p}p_{i,j}a_{i}-E_{q}\psi_{1}(x_{i})qa_{i,j}^{\prime }$$ (21)

(Model B2) Effect of cytosolic *X* (*x*_*i*_) on PIN1 amount (*p*) is incorporated by the replacement of *p* → *ψ*_1_(*x*_*i*_)*p* in Eq 14:

$$\frac{dp_{i,j}}{dt}=G_{p}\left(K\psi_{1}(x_{i})p\frac{\varphi_{a}(a_{i,j}^{\prime })}{\sum_{j}\varphi_{a}(a_{i,j}^{\prime })}-p_{i,j}\right)$$ (22)

(Model B3) Effect of cytosolic *X* (*x*_*i*_) on auxin synthesis (*A*) is incorporated by the replacement of *A* → *ψ*_1_(*x*_*i*_)*A* in Eq 11:

$$\frac{da_{i}}{dt}=G_{a}(\psi_{1}(x_{i})A-a_{i})-\sum_{j}f_{i,j}+\sum_{j}D_{a}(a_{i,j}^{\prime }-a_{i})$$ (23)

(Model B4) Effect of apoplast *X* ($x_{i,j}^{\prime }$) on efficiency of influx carrier (*E*_*q*_) is incorporated by the replacement of $E_{q}\to \psi_{2}(x_{i,j}^{\prime })E_{q}$ in Eq 13:

$$f_{i,j}=E_{p}p_{i,j}a_{i}-\psi_{2}(x_{i,j}^{\prime })E_{q}qa_{i,j}^{\prime }$$ (24)

(Model B5) Effect of apoplast *X* ($x_{i,j}^{\prime }$) on the efficiency of PIN1 efflux carrier function (*E*_*p*_) is incorporated by the replacement of $E_{p}\to \psi_{2}(x_{i,j}^{\prime })E_{p}$ in Eq 13:

$$f_{i,j}=\psi_{2}(x_{i,j}^{\prime })E_{p}p_{i,j}a_{i}-E_{q}qa_{i,j}^{\prime }$$ (25)

(Model B6) Effect of apoplast *X* ($x_{i,j}^{\prime }$) on PIN1 localization to cell membrane is incorporated by the replacement of $\varphi_{a}(a_{i,j}^{\prime })\to \varphi_{a}(a_{i,j}^{\prime })\varphi_{x}(x_{i,j}^{\prime })$ in Eq 14:

$$\frac{dp_{i,j}}{dt}=G_{p}\left(Kp\frac{\varphi_{a}(a_{i,j}^{\prime })\varphi_{x}(x_{i,j}^{\prime })}{\sum_{j}\varphi_{a}(a_{i,j}^{\prime })\varphi_{x}(x_{i,j}^{\prime })}-p_{i,j}\right)$$ (26)

*ψ*_1_(*x*_*i*_), $\psi_{2}(x_{i,j}^{\prime })$, and $\varphi_{x}(x_{i,j}^{\prime })$ are regulatory functions that depend on molecule *X*. The equations and regulatory functions used in numerical simulations are summarized in S1 Table.

#### Effect of symplast diffusion of molecule *X*

In Model B, we examined the symplast diffusion of molecule *X* (i.e., direct diffusion between neighboring cells; Fig 1F), instead of the simple diffusion between cytoplasm and apoplast, by replacing Eqs 18 and 19 with

$$\frac{dx_{i}}{dt}=G_{x}(\theta (a_{i})-x_{i})+\sum_{j}D_{x1}(x_{j}-x_{i})$$ (27)

$$\frac{dx_{i,j}^{\prime }}{dt}=-G_{x}x_{i,j}^{\prime }$$ (28)

where *G*_*x*_ and *θ*(*a*_*i*_) have the same notations as in Eqs 18 and 19, and *D*_*x*1_ is the diffusion coefficient between cells. Molecule *X* is synthesized depending on cytosolic auxin and degraded at a constant rate (the first terms of the right-hand sides of Eqs 27 and 28) and diffuses between cells (the second term of Eq 27). Equilibria of *x*_*i*_ and $x_{i,j}^{\prime }$ are given respectively by

$$x_{e}{}_{q}=\theta (a_{e}{}_{q})\;\mathrm{and}\;x_{eq}^{\prime }=0$$ (29)

#### Effect of apoplast diffusion of molecule

We also examined the apoplast diffusion of molecule *X* (i.e., direct diffusion between neighboring apoplast spaces; Fig 1F), instead of the simple diffusion, by replacing Eqs 18 and 19 with

$$\frac{dx_{i}}{dt}=G_{x}(\theta (a_{i})-x_{i})-KSx_{i}$$ (30)

$$\frac{dx_{i,j}^{\prime }}{dt}=-G_{x}x_{i,j}^{\prime }+\frac{S}{V}(x_{i}+x_{j})+\sum_{\left(k,l\right)}\frac{D_{x2}}{V}(x_{k,l}^{\prime }-x_{i,j}^{\prime })$$ (31)

where *G*_*x*_, *V*, and *θ*(*a*_*i*_) have the same notations as in Eqs 18 and 19, *K* is the number of neighboring cells, *S* is the secretion coefficient, *D*_*x*2_ is the diffusion coefficient between neighboring apoplast spaces, and apoplast (*k*, *l*) is a neighbor of apoplast (*i*, *j*). Molecule *X* is synthesized depending on cytosolic auxin and degraded at a constant rate (the first terms of the right-hand sides of Eqs 30 and 31), is secreted to apoplast spaces (the second terms), and diffuses between apoplast spaces (the third term of Eq 31). Equilibria of *x*_*i*_ and $x_{i,j}^{\prime }$ are given respectively by

$$x_{e}{}_{q}=G_{x}\theta (a_{e}{}_{q})/(G_{x}+KS)\;\mathrm{and}\;x_{eq}^{\prime }=2S\theta (a_{e}{}_{q})/V(G_{x}+KS)$$ (32)

### Numerical simulations

We used one-dimensional arrays of *N* = 200, 50, or 40 cells and two-dimensional sheets of 20 × 20 or 14 × 14 hexagonal cells in the numerical simulations. Initial values of auxin, PIN1, and molecule *X* are given by their equilibrium with 1.0% fluctuation. The numerical simulations were performed using the Euler method with time step Δ*t* = 0.001 under the periodic boundary condition. Equations and regulatory functions used are summarized in S1 Table. Parameter values used are described in figure legends.

### Index of spatial scale

To evaluate the spatial scale of auxin patterns generated by the numerical simulations, we used wavelength of auxin maxima (*L*_1_) and average size of auxin maximum (*L*_2_) as indices of the spatial scale (Fig 1G). In a one-dimensional array of *N* cells in the periodic boundary condition, auxin concentration of cell *n* or apoplast (*n*, *n*+1) is denoted here by *c*_*n*_, where *n* = 0,1,⋯,*N* and *c*_0_ ≡ *c*_*N*_.

#### Wavelength of auxin maxima pattern (*L*_1_)

After discrete Fourier transform of auxin concentration $c_{n}\;:\;F(k)=\frac{1}{N}\sum_{n=0}^{N-1}c_{n}e^{-i\frac{2\pi kn}{N}}\;(k=0,1,\cdots ,N-1)$, we determined wavenumber *k* = *k*_1_ ∈ [1,*N*/2] showing the largest spectral intensity of |*F*(*k*)|^2^ and then used the corresponding wavelength defined by

$$L_{1}\equiv N/k_{1}\in [2,N]$$ (33)

as an index of the spatial scale.

#### Average size of auxin maximum (*L*_2_)

A cell or an apoplast space with $c_{n}>\bar{c}$ is called an “auxin spot” and a successive string of auxin spots is denoted by an “auxin cluster” where $\bar{c}$ is the average of *c*_*n*_. The average size of auxin maximum (*L*_2_) is defined as the total number of auxin spots divided by that of auxin clusters.

## Results

### Model O

The phyllotaxis pattern of auxin maxima has been explained by a class of simplified mathematical models based on the feedback dynamics between auxin and PIN1. Because these models do not consider extracellular region (i.e., apoplast), auxin moves directly between cells by PIN1-dependent directional transport and passive diffusion to change its distribution (Fig 1A). On the other hand, PIN1 is asymmetrically localized to the cell membrane, preferentially toward neighboring cells with high auxin concentrations. We used Model O (Eqs 1–3), which is one of the simplest representations of such dynamics, to examine the spatial regularity control of auxin maxima pattern. As in previous reports [11, 12], we confirmed that Model O can form auxin maxima patterns with spatial regularity, an essential characteristic of phyllotaxis, focusing on its spatial scale.

#### Spatial regularity control in Model O

We considered a one-dimensional array of *N* cells under the periodic boundary condition in Model O (Eqs 1–3 with *K* = 2), and performed a linear stability analysis of the equilibrium (see S1 Text (ii) for detail). This theoretical analysis shows that eigenvalue *λ*_*k*_, which is associated with the growth rate of the pattern with wavenumber *k*(= 0,1,⋯,*N* − 1), is given by

$$\lambda_{k}(\nu )=4c_{2}\nu^{2}+2c_{1}\nu +c_{0}-2c_{2}$$ (34)

where *ν* ≡ cos(2*πk*/*N*) ∈ [−1,1], *c*_0_ ≡ −(*G*_*a*_ + 2*c*_1_ + 2*c*_2_), *c*_1_ ≡ *E*_*p*_*p* + *D*_*a*_, and $c_{2}\equiv -E_{p}pa_{eq}\varphi_{0}^{\prime }(a_{eq})/2\varphi_{0}(a_{eq})$. The condition for non-uniform patterns is described by

$$|\nu_{*}|<1\;\mathrm{and}\;\lambda_{k}\left(\nu_{*}\right)>0$$ (35)

where ν_*_ = −*c*_1_/4*c*_2_. When Eq 35 is satisfied (i.e., spatial homogeneity is broken), the spatial scale (i.e., wavelength L_*_) of the pattern with the highest growth rate depends on parameter values and is given by

$$L_{*}=2\pi /\cos^{-1}\left(\nu_{*}\right)\;(cells)$$ (36)

where L_*_ increases as ν_*_ becomes large.

**Fig 2:**
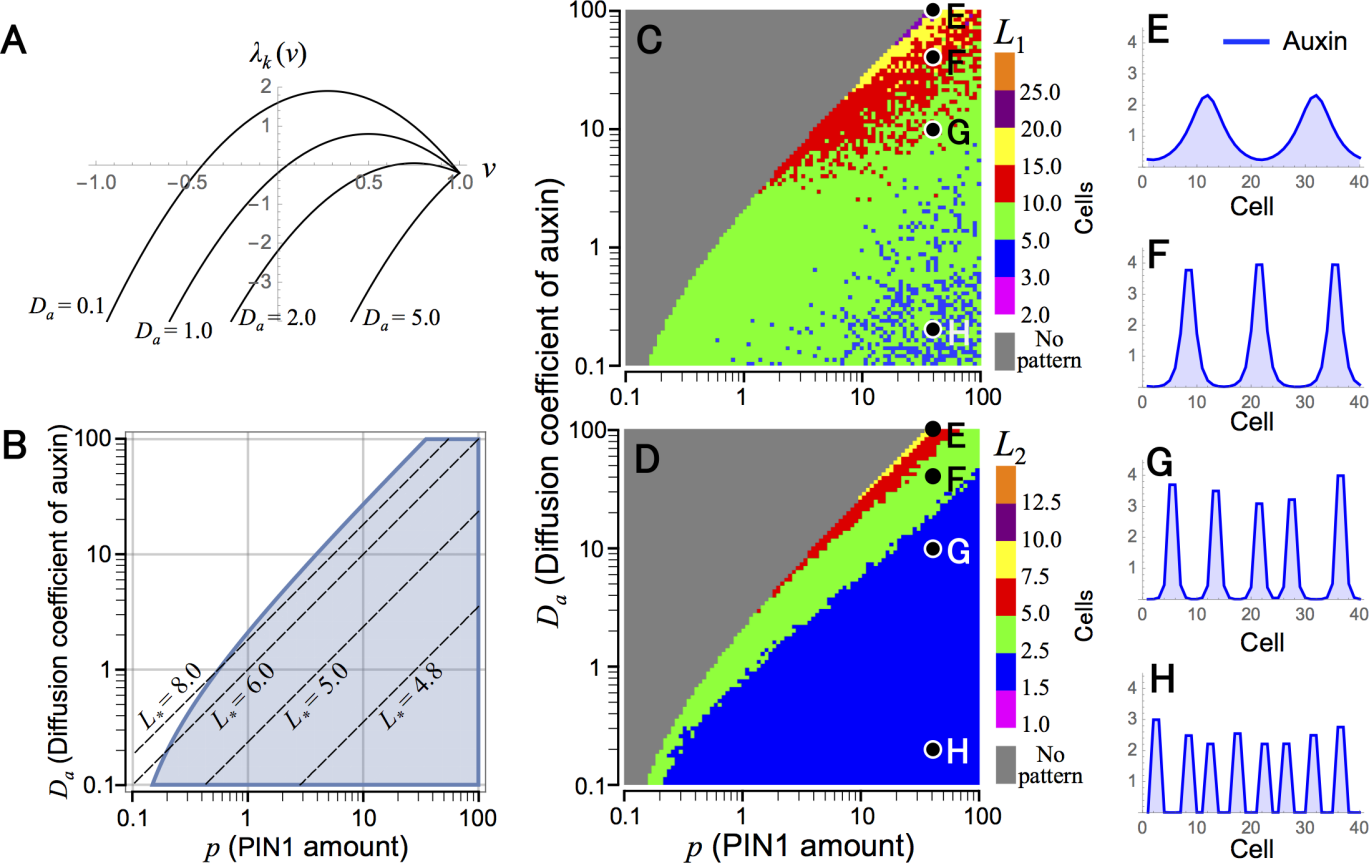
Spatial regularity of auxin pattern in Model O. (A) The eigenvalues are shown for continuous values of *ν* in different values of *D*_*a*_ (Eq 34). (B) The parameter condition that the equilibrium becomes unstable is indicated by the shaded area in the *p*−*D*_*a*_ plane (Eq 35). Broken lines indicate Eq 36 for different values of L_*_. (C and D) Wavelength of auxin maxima pattern (*L*_1_) (C) and average size of auxin maximum (*L*_2_) (D) were determined by numerical simulations in *p*−*D*_*a*_ plane. (E–H) Examples of auxin pattern indicated in C and D with parameter conditions of *p* = 40.0 and *D*_*a*_ = 100.0 (E), 40.0 (F), 10.0 (G), and 0.2 (H). Numerical simulations were performed in a one-dimensional array of *N* = 200 cells (C and D) or *N* = 40 cells (E–H) by the Euler method with time step Δ*t* = 0.001 under the periodic boundary condition. Initial values of variables were given by their equilibrium with 1.0% fluctuation. Eqs 1–3 and regulatory function *φ*_0_(*a*_*j*_) = *a*_*j*_^*n*^ are used with parameter values of *K* = 2, *A* = *E*_*p*_ = *G*_*p*_ = 1.0, *G*_*a*_ = 0.2, and *n* = 2.0 (A–H) and *p* = 1.0 (A).

**Fig 3:**
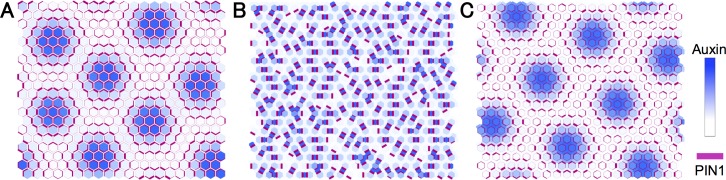
Examples of auxin pattern in two-dimensional space in Models O, A, and B6. Like the one-dimensional case, spatially regular patterns of auxin maxima can be generated in the two-dimensional space in Model O (A) and Model B6 (C), but cannot in Model A (B). Auxin concentration and PIN1 density are indicated in blue and by the thick magenta lines, respectively. Equations and regulatory functions are used as in S1 Table with parameter values of *K* = 6, *A* = *E*_*p*_ = *G*_*p*_ = 1.0, *D*_*a*_ = *p* = *n* = 2.0, *G*_*a*_ = 0.2, and *R* = 4.0 (A), *K* = 6, *A* = *E*_*p*_ = *E*_*q*_ = *G*_*p*_ = *D*_*a*_ = *V* = 1.0, *G*_*a*_ = 0.2, *p* = *q* = 5.0, and *R* = *n* = 3.0 (B), and *K* = 6, *A* = *E*_*p*_ = *E*_*q*_ = *G*_*p*_ = *G*_*x*_ = *D*_*a*_ = *D*_*x*_ = *V* = 1.0, *G*_*a*_ = 0.2, *p* = *q* = 5.0, and *R* = *m* = 3.0 (C). Numerical simulations were performed in two-dimensional sheets of 20 × 20 hexagonal cells by the Euler method with time step Δ*t* = 0.001 under the periodic boundary condition. Initial values of variables were given by their equilibrium with 1.0% fluctuation.

In the case of the regulatory function for PIN1 polarization *φ*_0_(*a*_*j*_) = *a*_*j*_^*n*^, Eq 35 and ν_*_ become

$$D_{a}<(2n-1)E_{p}p-\sqrt{2nG_{a}E_{p}p}\;\mathrm{and}$$ (37)

$$\nu_{*}=\left(1+R_{a}\right)/2n,$$ (38)

respectively, where *R*_*a*_ ≡ *D*_*a*_/*E*_*p*_*p* corresponds to the strength ratio of auxin diffusion to transport by PIN1. Eigenvalue *λ*_*k*_(*ν*) (Eq 34) and the condition for pattern formation (Eq 37) are shown in Fig 2A and 2B, respectively. This result predicts that the spatial scale of formed patterns L_*_ becomes large as the diffusion coefficient (*D*_*a*_) increases or auxin transport by PIN1 (*E*_*p*_*p*) and the regulatory strength of PIN1 polarization (*n*) decrease. This prediction is supported by numerical simulations, in which non-uniform auxin distribution is observed in the parameter area corresponding to Eq 37 (Fig 2B), and the wavelength of auxin pattern (*L*_1_) and average size of auxin maximum (*L*_2_) increase by decreasing *p* or increasing *D*_*a*_ (Fig 2C–2H). Besides, Model O can also generate regular patterns of auxin maxima in the two-dimensional space (Fig 3A). These results are consistent with previous reports [11, 12]. Therefore, Model O can reproduce the regular distance between auxin maxima, which is an essential characteristic of phyllotaxis pattern, suggesting that “up-the-gradient” is a central concept of this pattern formation.

### Model A

#### Incorporation of extracellular space

While Model O is suitable for understanding the “up-the-gradient” concept as described in the previous section, it is not appropriate when considering cellular spatial structures because plant cells are separated from each other by the extracellular region (i.e., apoplast). Thus, we investigated how the introduction of extracellular region affects the spatial regularity of auxin maxima. In the revised model called Model A (Eqs 11–14), auxin moves between cytoplasm and apoplast; outwardly by PIN1, inwardly by influx carriers, and non-directionally by passive diffusion, instead of the direct migration between cells in Model O (Fig 1B). On the other hand, PIN1 is asymmetrically localized to the cell membrane depending on the auxin concentration of neighboring apoplast spaces, instead of that of neighboring cells in Model O.

#### Spatial regularity control in Model A

We consider a one-dimensional array of *N* cells that are separated from each other by apoplast space under the periodic boundary condition in Model A (Eqs 11–14 with *K* = 2), and we performed a linear stability analysis of the equilibrium as in Model O (see S1 Text (iii) for detail). This theoretical analysis shows that eigenvalue *λ*_*k*_, which is associated with the growth rate of the pattern with wavenumber *k*(= 0,1,⋯,*N* −1), is given by

$$\lambda_{k}(\nu )=2c_{1}\nu +c_{0}$$ (39)

where *ν* ≡ cos(2*πk*/*N*) ∈ [−1,1], *c*_0_ ≡ *α* + *β* − (2(*E*_*q*_*q* + *D*_*a*_)/*V* + *G*_*a*_), *c*_1_ ≡ (*α* − *β*)/2, *α* ≡ 2(*E*_*p*_*p* + *D*_*a*_)(*E*_*q*_*q* + *D*_*a*_)/*V*(2*E*_*p*_*p* + 2*D*_*a*_ + *G*_*a*_), and $\beta \equiv E_{p}p(2(E_{q}q+D_{a})a_{eq}^{\prime }+G_{a}A)\varphi_{a}^{\prime }(a_{eq}^{\prime })/V(2E_{p}p+2D_{a}+G_{a})\varphi_{a}(a_{eq}^{\prime })$. It is also shown that the condition for generating spatial heterogeneity is described by

$$\lambda_{k}(-1)>0$$ (40)

When Eq 40 is satisfied (i.e., spatial homogeneity is broken), the spatial scale (i.e., wavelength L_*_) of the pattern with the highest growth rate (i.e., the largest *λ*_*k*_) is independent of parameter values and always given by

$$L_{*}=2\;(apoplast\;spaces)$$ (41)

This theoretical result indicates that the pattern of apoplast spaces alternating between high and low auxin concentrations always grows fastest compared to that with longer spatial scales and is consistent with the result reported by Sahlin *et al*. [16]. This also indicates that the extracellular space has a destructive effect on the spatial regularity of auxin maxima.

In the case of regulatory function for PIN1 polarization $\varphi_{a}(a_{i,j}^{\prime })={\left(a_{i,j}^{\prime }\right)}^{n}$, Eq 40 becomes

$$D_{a}<(n-1)E_{p}p$$ (42)

**Fig 4:**
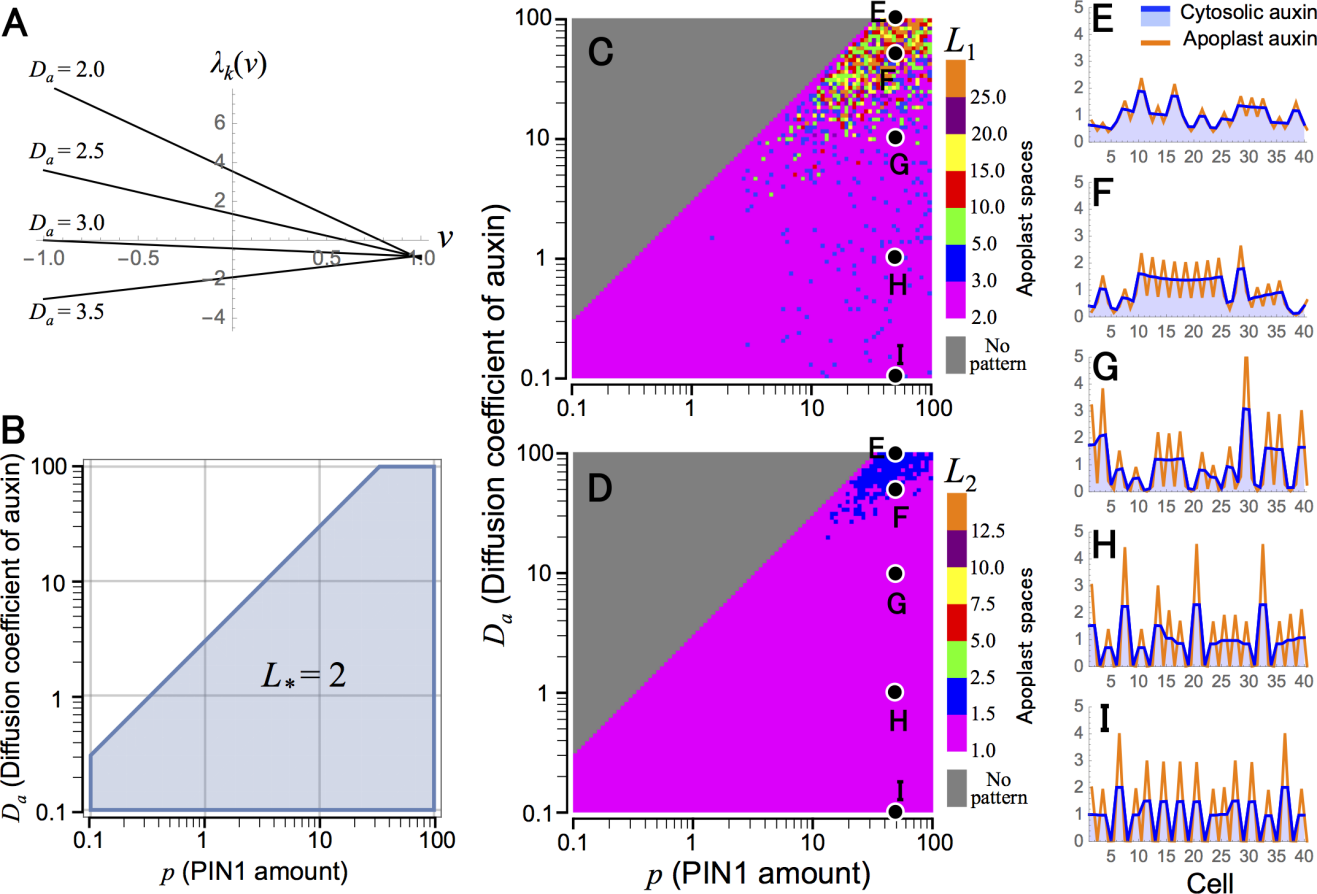
Spatial regularity of auxin pattern in Model A. (A) The eigenvalues are shown for continuous values of *ν* in different values of *D*_*a*_ (Eq 39). (B) The parameter condition that the equilibrium becomes unstable is indicated by the shaded area in the *p*−*D*_*a*_ plane (Eq 42). (C and D) Wavelength of auxin maxima pattern (*L*_1_) (C) and average size of auxin maximum (*L*_2_) (D) were determined by numerical simulations in *p*−*D*_*a*_ plane. (E–I) Examples of auxin pattern with parameter conditions indicated in C and D. Numerical simulations were carried out in a one-dimensional array of *N* = 200 cells (C and D) or *N* = 40 cells (E–I), which are separated from each other by apoplast space, by the Euler method with time step Δ*t* = 0.001 under the periodic boundary condition. Initial values of variables were given by their equilibrium with 1.0% fluctuation. Eqs 11–14 and regulatory function $\varphi_{a}(a_{i,j}^{\prime })={\left(a_{i,j}^{\prime }\right)}^{n}$ are used with parameter values of *K* = 2, *A* = *E*_*p*_ = *E*_*q*_ = *G*_*p*_ = *V* = 1.0, *q* = 10.0, *G*_*a*_ = 0.2, and *n* = 4.0 (A–I), *p* = 1.0 (A) or 50.0 (E–I), and *D*_*a*_ = 100.0 (E), 50.0 (F), 10.0 (G), 1.0 (H), or 0.1 (I).

Eigenvalue *λ*_*k*_(*ν*) (Eq 39) and the condition for pattern formation (Eq 42) are shown in Fig 4A and 4B, respectively. These theoretical results are consistent with numerical simulations, in which a non-uniform distribution of auxin occurs in the corresponding parameter region of Eq 42 (Fig 4B–4D). On the other hand, opposite to Model O, the spatial scale of formed patterns becomes extremely small in most parameter conditions examined. In addition, similar results are observed in the case of two-dimensional space, in which spatially regular patterns of auxin maxima seen in Model O are completely eliminated in Model A (Fig 3B).

#### Effect of symplast diffusion of auxin

**Fig 5:**
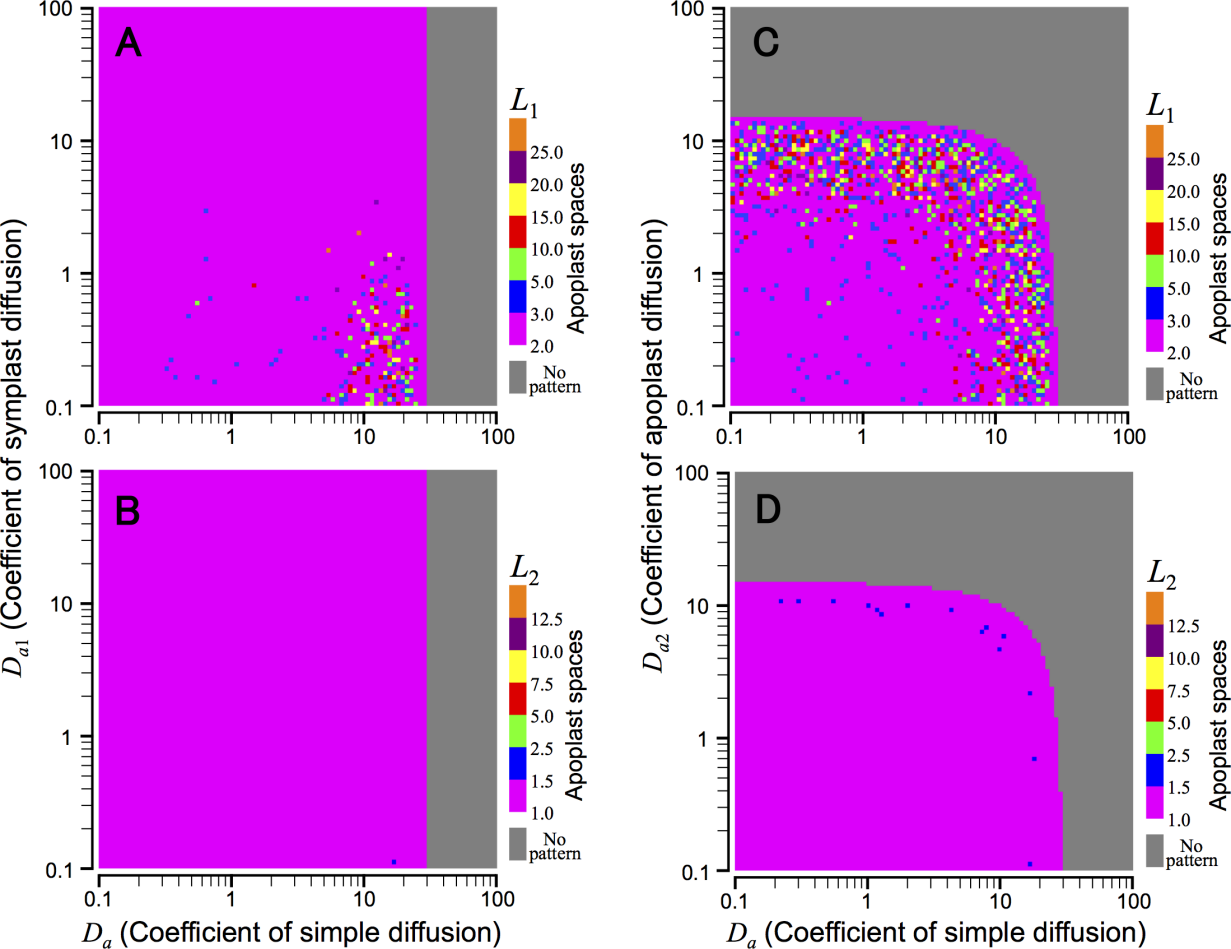
Effects of symplast and apoplast diffusions in Model A. Wavelength of auxin maxima pattern (*L*_1_) (A and C) and average size of auxin maximum (*L*_2_) (B and D) were determined in the presence of the symplast diffusion (A and B, Eqs 12–14 and 16) or apoplast diffusion (C and D, Eqs 11, 13, 14 and 17), in addition to the simple diffusion between cytoplasm and apoplast (Fig 1F). Numerical simulations were performed in a similar manner as in Fig 4 with parameter values of *K* = 2, *A* = *E*_*p*_ = *E*_*q*_ = *G*_*p*_ = *V* = 1.0, *q* = 10.0, *G*_*a*_ = 0.2, and *n* = 4.0 (A–D). Equations and regulatory functions used are summarized in S1 Table.

In Model A described, we consider auxin diffusion between cytoplasm and apoplast (Fig 1F, simple diffusion). However, because auxin can directly move between cells through plasmodesmata [22–24] (Fig 1F, symplast diffusion), we examined the effect of the symplast diffusion on pattern formation by replacing Eq 11 with Eq 16. Numerical simulations show that patterns with larger spatial scales cannot be recovered under this diffusion condition (Fig 5A and 5B).

#### Effect of apoplast diffusion of auxin

In plant tissues, the apoplast spaces are connected to each other and auxin can move freely among them (Fig 1F, apoplast diffusion). Thus, we also examined the effect of the apoplast diffusion by replacing Eq 12 with Eq 17. Linear stability analysis predicts that this change causes no essential effects on the spatial regularity of the auxin maxima pattern (S1 Text (iv)). In fact, this theoretical prediction is supported by numerical simulations, in which the spatial scale of the generated patterns remains extremely small as in Fig 4 (Fig 5C and 5D).

### Model B

#### Incorporation of diffusible molecule

The previous section showed that extracellular space has a disruptive effect on the spatial regularity of auxin maxima. This result strongly suggests that phyllotaxis pattern cannot be explained by the “up-the-gradient” framework alone but requires an unknown molecular mechanism. On the other hand, the spatial regularity can be generated even in the presence of extracellular space if PIN1 polarization depends on the auxin concentration of neighboring cells [16], suggesting a mechanism that transmits the auxin concentration between neighboring cells. This could be fulfilled by considering a factor that is induced by auxin and diffuses freely. To verify this possibility, we assumed a molecule *X* that is expressed depending on auxin concentration and diffuses between cytoplasm and apoplast (Fig 1C). The framework of the revised model called Model B is obtained by incorporating molecule *X* into Model A, and then we considered various feedback effects of *X* on auxin–PIN1 dynamics as follows (Fig 1D):

(Model B1) Cytosolic *X* (*x*_*i*_) affects the amount of auxin influx carrier (*q*).

(Model B2) Cytosolic *X* (*x*_*i*_) affects the PIN1 amount (*p*).

(Model B3) Cytosolic *X* (*x*_*i*_) affects the auxin synthesis (*A*).

(Model B4) Apoplast *X* ($x_{i,j}^{\prime }$) affects the efficiency of auxin influx carrier function (*E*_*q*_).

(Model B5) Apoplast *X* ($x_{i,j}^{\prime }$) affects the efficiency of PIN1 function (*E*_*p*_).

(Model B6) Apoplast *X* ($x_{i,j}^{\prime }$) affects the PIN1 localization to cell membrane.

**Fig 6:**
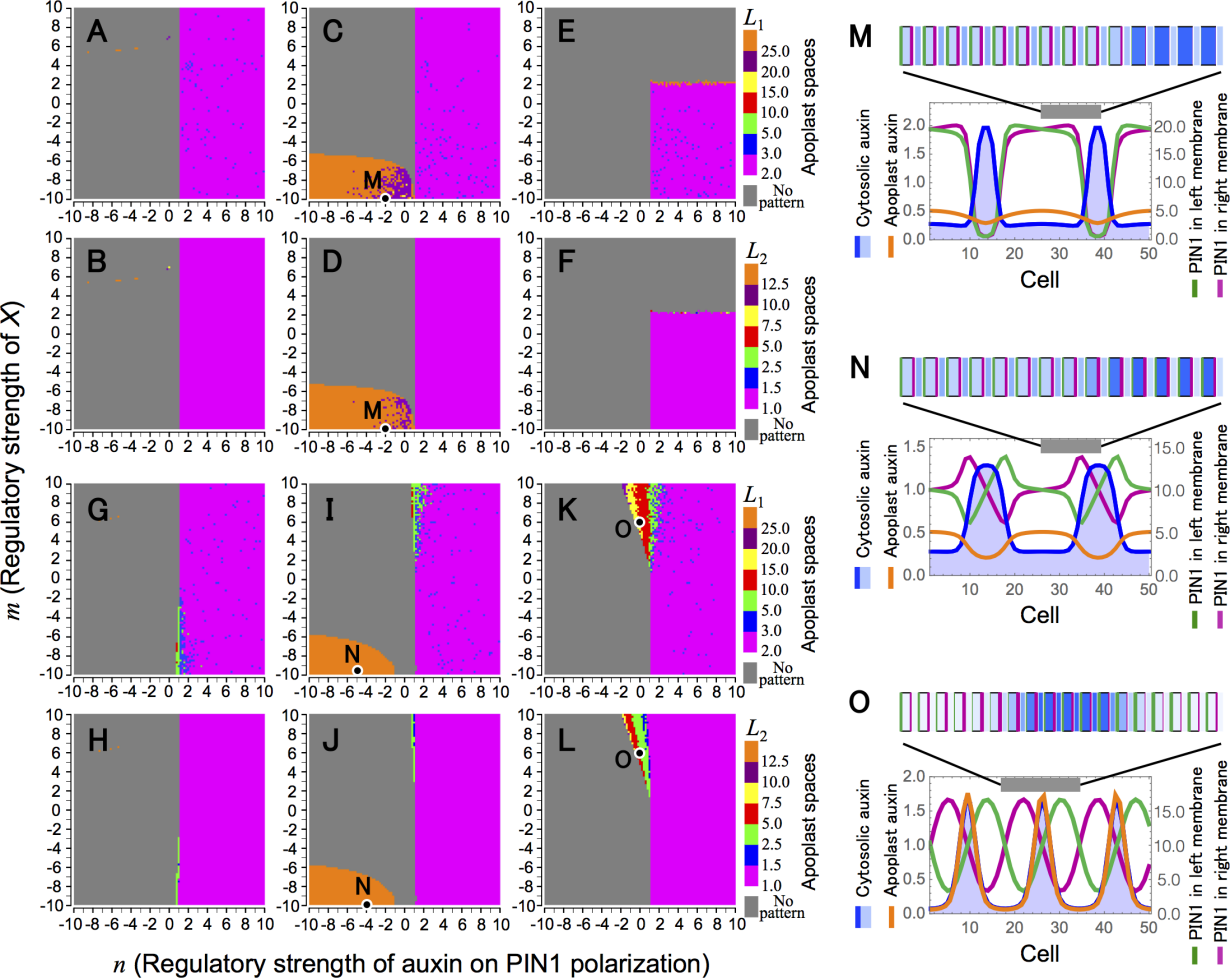
Spatial regularity of auxin pattern in Model B. (A–L) Wavelength of auxin maxima pattern (*L*_1_) (A, C, E, G, I, and K) and average size of auxin maximum (*L*_2_) (B, D, F, H, J, and L) were determined in Model B1 (A and B), Model B2 (C and D), Model B3 (E and F), Model B4 (G and H), Model B5 (I and J), and Model B6 (K and L). (M–O) Examples of regular patterns with parameter conditions indicated in C and D (M; Model B2 with *n* = −2.0 and *m* = −10.0), in I and J (N; Model B5 with *n* = −4.0 and *m* = −10.0), and in K and L (O; Model B6 with *n* = 0.0 and *m* = 6.0). Numerical simulations were performed in a one-dimensional array of *N* = 200 (A–L) or 50 (M–O) cells, which are separated each other by apoplast space, by the Euler method with time step Δ*t* = 0.001 under the periodic boundary condition. Initial values of variables were given by their equilibrium with 1.0% fluctuation. Equations and regulatory functions used are summarized in S1 Table with parameter values of *K* = 2, *A* = *E*_*p*_ = *E*_*q*_= *G*_*x*_ = *G*_*p*_ = *D*_*a*_ = *V* = *r* = 1.0, *p* = *q* = *D*_*x*_ = 10.0, and *G*_*a*_ = 0.2 (A–O).

Models B1–B6 are described in detail in the Model section, and equations and regulatory functions are summarized in S1 Table. To examine whether each feedback regulation can restore the spatial regularity of the auxin pattern as in Model O, we carried out numerical simulations with systematically changing regulatory strengths of apoplast auxin on PIN1 polarization (*n*) and of molecule *X* (*m*) (Fig 6). In the one-dimensional space, we found three conditions for regular pattern formation: negative values of *m* in Models B2 (Fig 6C, 6D and 6N) and B5 (Fig 6I, 6J, and 6N) and positive values of *m* in Model B6 (Fig 6K, 6L, and 6O). However, the former two do not appear to be plausible because no regular patterns are formed under the corresponding conditions in the two-dimensional space (S1 and S2 Figs). By contrast, in both one- and two-dimensional spaces, only Model B6 generates auxin maxima with relatively large wavelengths, which depend on parameter values (Fig 5K and 5L). This result suggests that the feedback via PIN1 polarization is essential for the auxin maxima pattern.

#### Effect of diffusion variation in Model B

In addition to the diffusion between cytoplasm and apoplast, signal molecules in plants have two major diffusion types (Fig 1F): (i) direct diffusion between cells (symplast diffusion) [19–21] and (ii) diffusion among extracellular spaces (apoplast diffusion) [25, 26] as described in the above. We examined how these diffusion types affect the spatial regularity. Symplast or apoplast diffusion can be incorporated into Model B by replacing Eqs 18 and 19 with Eqs 27 and 28 or with Eqs 30 and 31, respectively (S1 Table). These diffusion types provide numerical simulations similar to those in the simple diffusion (Fig 6), in which normal auxin maxima patterns can be restored only in the feedback regulation of PIN1 polarization (Model B6) but not in the other conditions (Models B1–B5) (S3 Fig). This result reconfirms that the feedback from auxin to PIN1 polarization is crucial for phyllotaxis pattern whereas the diffusion type of the diffusible molecule is not. Next, we investigated Model B6 in detail.

#### Spatial regularity control in Model B6

In Model B6, regular patterns of auxin maxima can be generated even in the absence of the feedback from apoplast auxin to PIN1 polarization (Figs 5K and 5L and S3K and S3L; *n* = 0). Accordingly, this feedback regulation is not essential and is not considered in the following analysis (Fig 1E; Eqs 11–13, 18, 19 and 26 with *K* = 2 and $\varphi_{a}(a_{i,j}^{\prime })=1$). As with Model A, we consider a one-dimensional array of *N* cells and performed a linear stability analysis of the equilibrium (see S1 Text (v) for detail). By a similar argument to that of Model O, we can obtain approximate equations corresponding to Eqs 34–36:

$$\lambda_{k}(\nu )\approx 4c_{2}\nu^{2}+2c_{1}\nu +c_{0}-2c_{2}$$ (43)

$$|\nu_{*}|<1\;and\;\lambda_{k}(\nu_{*})>0$$ (44)

$$L_{*}\approx 2\pi /\cos^{-1}\left(\nu_{*}\right)\;(cells)$$ (45)

where *ν* ≡ cos(2*πk*/*N*) ∈ [−1,1], ν_*_ ≈ −*c*_1_/4*c*_2_, *c*_0_ ≡ 2*c*_1_ − 2*c*_2_ − (2*E*_*p*_*p* + 2*D*_*a*_ + *G*_*a*_), *c*_1_ ≡ *γ*(*E*_*p*_*p* + *D*_*a*_), $c_{2}\equiv -\gamma \kappa E_{p}pa_{eq}\theta^{\prime }(a_{eq})\varphi_{x}^{\prime }(x_{eq}^{\prime })/2\varphi_{x}(x_{eq}^{\prime }),$ *γ* ≡ (*E*_*q*_*q* + *D*_*a*_)/(2*E*_*q*_*q* + 2*D*_*a*_ + *VG*_*a*_), and *κ* ≡ *D*_*x*_*G*_*x*_/(2*D*_*x*_ + *G*_*x*_)(2*D*_*x*_ + *VG*_*x*_) Eqs 43–45 are associated with the pattern growth rate, condition for generating spatial heterogeneity, and spatial scale of generated patterns, respectively. This theoretical result indicates that spatial scale L_*_ changes depending on parameter values as in Model O, and thus the spatial regularity of auxin pattern can be restored in Model B6.

**Fig 7:**
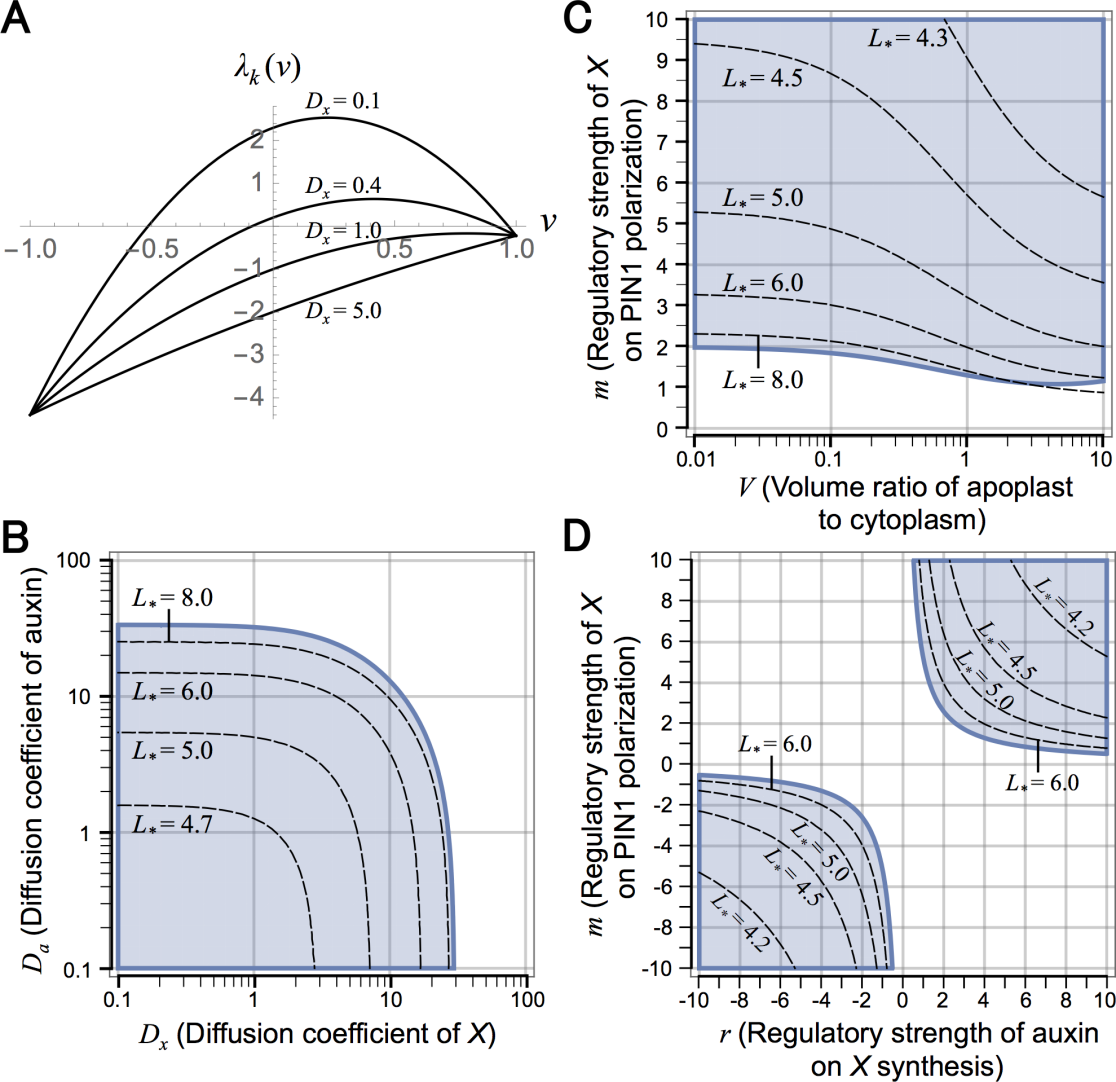
Spatial regularity control in Model B6. (A) The eigenvalues are shown for continuous values of *ν* in different values of *D*_*x*_ (Eq 43). (B–D) The parameter condition that the equilibrium becomes unstable is shown by shaded area in *D*_*x*_−*D*_*a*_ (B), *V*−*m* (C), and *r*−*m* (D) planes (Eq 44). Broken lines indicate Eq 45 for different values of L_*_. Regulatory functions as in Fig 8 (*θ*(*a*_*i*_) = 2*a*_*i*_^*r*^/*a*_*eq*_^*r*^ + *a*_*i*_^*r*^), $\varphi_{a}(a_{i,j}^{\prime })=1$, and $\varphi_{x}(x_{i,j}^{\prime })={\left(x_{i,j}^{\prime }\right)}^{m}$) are used with parameter values of *K* = 2, *A* = *E*_*p*_ = *E*_*q*_ = 1.0, and *G*_*a*_ = 0.2 (A–D), *p* = *q* = 2.0 (A) or 10.0 (B–D), *D*_*a*_ = 0.1 (A) or 1.0 (C and D), *D*_*x*_ = 5.0 (C and D), *G*_*x*_ = 0.5 (A), 1.0 (B), or 5.0 (C and D), *V* = 0.1 (A) or 1.0 (B and D), *r* = 2.0 (A and B) or 4.0 (C), and *m* = 6.0 (A) or 5.0 (B).

**Fig 8:**
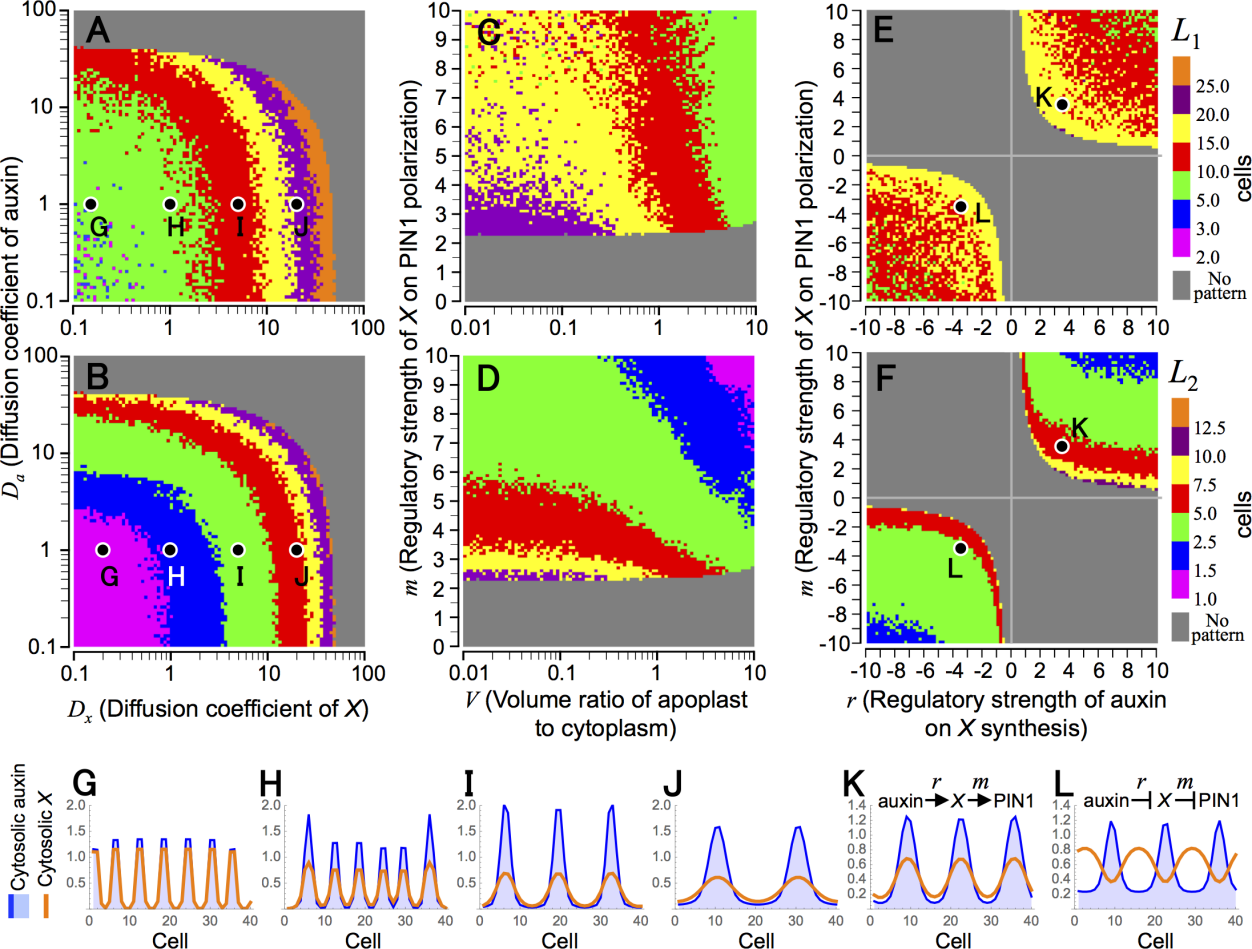
Spatial regularity of auxin pattern in Model B6. (A–F) Wavelength of auxin maxima pattern (*L*_1_) (A, C, and E) and average size of auxin maximum (*L*_2_) (B, D, and F) were determined by numerical simulations in *V*−*m* (A and B), *D*_*x*_−*D*_*a*_ (C and D), and *r*−*m* (E and F) planes. (G–L) Examples of auxin pattern with parameter conditions indicated in A and B (G–J; *D*_*x*_ = 0.2, 1.0, 5.0, and 20.0, respectively, and *D*_*a*_ = 1.0) and in E and F (K and L; *r* = *m* = 3.5 and −3.5, respectively). Numerical simulations were carried out in a one-dimensional array of *N* = 200 cells (A–F) or *N* = 40 cells (G–L), which are separated from each other by apoplast space, by the Euler method with time step Δ*t* = 0.001 under the periodic boundary condition. Initial values of variables were given by their equilibrium with 1.0% fluctuation. Eqs 11–13, 18, 19 and 26. Regulatory functions *θ*(*a_i_*) = 2*a_i_^r^*/(*a_eq_^r^* + *a_i_^r^*), $\varphi_{a}(a_{i,j}^{\prime })=1$, and $\varphi_{x}(x_{i,j}^{\prime })={\left(x_{i,j}^{\prime }\right)}^{m}$ are used with parameter values of *K* = 2, *A* = *E*_*p*_ = *E*_*q*_ = *G*_*p*_ = *V* = 1.0, *p* = *q*= 10.0, *G*_*a*_ = 0.2, *G*_*x*_ = 0.5 (A and B) or 1.0 (C–F), *D*_*a*_ = 1.0 (C–F), *D*_*x*_ = 10.0 (C and D) or 20.0 (E and F), *r* = 2.0, and *m* = 6.0.

In the case of regulatory functions *θ*(*a*_*i*_) = 2*a*_*i*_^*r*^/(*a*_*eq*_^*r*^ + *a*_*i*_^*r*^) and $\varphi_{x}(x_{i,j}^{\prime })={\left(x_{i,j}^{\prime }\right)}^{m}$, ν_*_ becomes

$$\nu_{*}\approx \frac{2(1+R_{a})}{rm}\frac{\left(1+2R_{x})(V+2R_{x}\right)}{V+2(1+V)R_{x}}$$ (46)

where *R*_*a*_ ≡ *D*_*a*_/*E*_*p*_*p* and *R*_*x*_ ≡ *D*_*x*_/*G*_*x*_. Eigenvalue *λ*_*k*_(*ν*) (Eq 43) and the condition for pattern formation (Eq 44) are shown in Fig 7A and 7B–7D, respectively. Eqs 45 and 46 suggests that the spatial scale (L_*_) increases as diffusion coefficients of auxin (*D*_*a*_) and molecule *X* (*D*_*x*_) become large (Fig 7B), and this is consistent with numerical simulations (Fig 8A, 8B and 8G–8J). However, *X* diffusion is essential for pattern formation whereas auxin diffusion is not, because auxin maxima can be formed in the absence of auxin diffusion (*D*_*a*_ = 0) but cannot without *X* diffusion (*D*_*x*_ = 0) (S4 Fig). In plant tissues, the extracellular region is usually a very small space compared with cytoplasm, indicating that *V*, the volume ratio of apoplast to cytoplasm, is sufficiently small (i.e., *V* ≪ 1). Eq 46 also suggests that ν_*_, accordingly L_*_, increases as *V* becomes small and has the limit: lim_*V*→0_ ν_*_ ≈ 2(1 + *R*_*a*_)(1 + 2*R*_*x*_)/*rm* (Fig 7C). This is also consistent with numerical simulations (Fig 8C and 8D).

On the other hand, *r* and *m* are related to the regulatory strengths of auxin on *X* synthesis and of *X* on PIN1 polarization, respectively. Stable patterns are theoretically predicted and numerically generated in two separate parameter areas of *r*,*m* > 0 and *r*,*m* < 0 (Figs 7D, 8E and 8F), in which the two regulations are both stimulatory and inhibitory effects, respectively, leading to in-phase and anti-phase, respectively, patterns of auxin and molecule *X* (Fig 8K and 8L). This suggests that regular patterns are induced by stimulating PIN1 polarization by auxin while the way in which individual reactions are controlled is not so important.

## Discussion

In phyllotaxis pattern formation, auxin maxima involved in leaf primordia are formed in a self-organizing manner while maintaining a constant distance from each other. However, the molecular mechanism generating the spatial regularity remains unclear. This spatial regularity has been explained by simple mathematical models (Model O; Figs 1A, 2 and 3A), in which PIN1 is polarized preferentially toward neighboring cells with higher auxin concentrations [11–13]. But these models have two major problems concerning spatial structure: one is the absence of extracellular space and the other is how cells perceive auxin concentrations of neighbors for PIN1 polarization.

In this study, therefore, we intensively investigated how the spatial regularity of the phyllotaxis pattern is controlled under appropriate conditions for plant cells. We showed theoretically and numerically that auxin maxima patterns with large spatial scale are completely eliminated by introducing extracellular space (Model A; Figs 1B, 3B, 4 and 5). This strongly suggests that phyllotaxis pattern requires an unknown molecular mechanism as well as auxin–PIN1 mutual interaction. Furthermore, we found that regular patterns can be restored by the simple and plausible assumption that a diffusible molecule mediates the feedback from auxin to PIN1 polarization (Model B6; Figs 3C and 8). Although we mostly investigated in one-dimensional space, the same can be applied to the case of two-dimensional space. Model O can generate regular patterns of auxin maxima (Fig 3A). This spatial regularity is completely disrupted by considering extracellular space in Model A (Fig 3B), but is restored by introducing a diffusible molecule in Model B6 (Fig 3C). This diffusible molecule plays the role of transmitting auxin concentration to neighboring cells.

Auxin reportedly enhances the PIN1 localization at the cell membrane [27–29]. AUXIN-BINDING PROTEIN 1 (ABP1) might act as an apoplastic auxin receptor in the signaling pathway of PIN1 polarization although the function of ABP1 has recently contended [28, 30–32]. However, ABP1 probably makes no contribution to the phyllotaxis pattern formation regardless of whether it is an actual auxin sensor or not, because our study strongly suggests that the auxin maxima pattern cannot be established by the direct regulation of auxin on PIN1 polarization (Figs 4–6 and S1–S3).

Although our study showed that a diffusible factor can restore regular patterns that are disrupted by the presence of extracellular space, this finding does not rule out other possibilities for the spatial communication between cells. One such possible mechanism is mechanical force, including stress and strain, which affects the morphogenesis of plants and animals [33–36]. Mechanical force could stabilize the outgrowth of leaf primordia by feedback mechanism in which mechanical stress induces alignment of microtubules, enhancing cell elongation and primordial outgrowth, which reinforces the stress field [37]. In contrast, experimental evidence that mechanical force is involved during auxin maxima formation has not yet been obtained [4, 7]. However, auxin could alter the mechanical properties of the extracellular matrix by inducing cell-wall loosening [5, 6, 38, 39], suggesting that mechanical force may contribute to the pattern formation.

Our model predicts that the spatial scale of generated patterns (L_*_) becomes large by increasing ν_*_, which follows ν_*_ ∝ (1 + *R*_*a*_)/*a*_*eq*_|*θ*′(*a*_*eq*_)|, where *R*_*a*_ ≡ *D*_*a*_/*E*_*p*_*p* and *θ*(*a*_*i*_) is the regulatory function of auxin on *X* synthesis (Model B6; S1 Text, Eq 44). Therefore, if molecule *X* predicted in this paper exists, L_*_ is affected by the regulatory activity of auxin on the expression of *X* as well as by the amount of PIN1. That is, under the conditions of a constant amount of PIN1 and constant activity of PIN1 recycling between cytosol and cell membrane, it is expected that, as the gene expression control by auxin becomes weak, the spacing between auxin maxima gradually increases and patterns suddenly disappear under a threshold of the control strength. This prediction could be used to experimentally validate whether or not our model is correct. Auxin affects the expression of many genes by cooperating with the TRANSPORT INHIBITOR RESISTANT 1/AUXIN SIGNALING F-BOX (TIR1/AFB) F-box proteins, the AUXIN/INDOLE-3-ACETIC ACID (Aux/IAA) transcriptional coregulators, and sequence-specific binding proteins called AUXIN RESPONSE FACTORs (ARFs) [40–42]. Because these factors are possible candidates that control the expression of molecule *X*, our model could be verified using plants showing various expression activities by genetically manipulating these factors.

Our study could predict a diffusible factor that is essential for phyllotaxis pattern but remains to be found. This factor(s) *X* must satisfy the following requirements:

(i) *X* expression is regulated by auxin concentration.
(ii) *X* diffuses freely independent from PIN1 polarization.
(iii) *X* affects PIN1 polarization in the cell membrane.
(iv) The complete defect of *X* results in that of auxin maxima and accordingly that of leaf primordia.

Although such factors like *X* are not yet known, several diffusible molecules affecting PIN1 polarization have been reported. Strigolactone is a mobile plant hormone and cooperates with auxin to control shoot branching of plants. Auxin positively regulates the transcript of strigolactone biosynthesis genes and, in turn, strigolactone signaling triggers PIN1 depletion from the plasma membrane [43–45]. On the other hand, GOLVEN (GLV) genes encode small secretory peptides that are involved in root gravitropic responses and meristem organization in *Arabidopsis*. Transcription of GLV genes is rapidly induced by auxin, and the GLV peptide treatment stimulates the localization of auxin efflux carrier PIN2 at the cell membrane [46, 47]. Another mobile plant hormone cytokinin plays important roles in various developmental events through crosstalk with other plant hormones including auxin [48–50]. For example, in vascular differentiation, cytokine affects the orientation of PIN proteins in cell membrane while auxin regulates cytokinin signaling [51, 52]. Besides, also during lateral root organogenesis, cytokinin enhances the PIN1 depletion from cell membrane to affect PIN1 polarization [53–55]. Furthermore, it is reported that the localization of PIN proteins is affected by diffusible molecules such as jasmonate and narciclasine [56, 57]. It is not yet clear whether these molecules are involved in the phyllotaxis pattern or not. However, in near future, we hope that a molecule predicted theoretically in this study will be revealed experimentally.

## Supporting information

S1 Text Linear stability analysis. (PDF)

S1 Table Summary of equations and regulatory functions used in numerical simulations. (PDF)

S1 Fig Auxin pattern in two-dimensional space in Model B2. Auxin concentration and PIN1 density are indicated in blue and by the thick magenta lines, respectively. Equations and regulatory functions are described in S1 Table with parameter values of *K* = 6, *A* = *E*_*p*_ = *E*_*q*_ = *G*_*p*_ = *G*_*x*_ =*D*_*a*_ = *V* = *r* = 1.0, *G*_*a*_ = 0.2, and *p* = *q* = *D*_*x*_ = 10.0. Numerical simulations were performed in two-dimensional sheets of 14 × 14 hexagonal cells by the Euler method with time step Δ*t* = 0.001 under the periodic boundary condition. Initial values of variables were given by their equilibrium with 1.0% fluctuation.
(EPS)

S2 Fig Auxin pattern in two-dimensional space in Model B5. Auxin concentration and PIN1 density are indicated in blue and by the thick magenta lines, respectively. Equations and regulatory functions are described in S1 Table with parameter values of *K* = 6, *A* = *E*_*p*_ = *E*_*q*_ = *G*_*p*_ = *G*_*x*_ =*D*_*a*_ = *V* = *r* = 1.0, *G*_*a*_ = 0.2, and *p* = *q* = *D*_*x*_ = 10.0. Numerical simulations were carried out in two-dimensional sheets of 14 × 14 hexagonal cells by the Euler method with time step Δ*t* = 0.001 under the periodic boundary condition. Initial values of variables were given by their equilibrium with 1.0% fluctuation.
(EPS)

S3 Fig Effect of diffusion variation in Model B. Wavelength of auxin maxima pattern (*L*_1_) (A, C, E, G, I, and K) and average size of auxin maximum (*L*_2_) (B, D, F, H, J, and L) were determined in Model B1 (A and B), Model B2 (C and D), Model B3 (E and F), Model B4 (G and H), Model B5 (I and J), and Model B6 (K and L). The symplast diffusion (A–F) or apoplast diffusion (G–L) of molecule *X* was used instead of the simple diffusion between cytoplasm and apoplast in Fig 5 (Fig 1F). Numerical simulations were carried out in a similar manner as shown in Fig 6. Equations and regulatory functions are used as in S1 Table with parameter values of *K* = 2, *A* = *E*_*p*_ = *E*_*q*_ = *G*_*x*_ = *G*_*p*_ =*D*_*a*_ = *V* = *r* = 1.0, *p* = *q* = 10.0, and *G*_*a*_ = 0.2 (A–L), *D*_*x*1_ = 10.0 (A–F), *D*_*x*2_ = 10.0 (G–L), and *S* = 1.0 (G–L).
(TIFF)

S4 Fig Effect of the absence of auxin diffusion or *X* diffusion in Model B6. Examples of auxin distribution in the absence of auxin diffusion (*D*_*a*_ = 0.0; A–E) or *X* diffusion (*D*_*x*_ = 0.0; F–J) in Model B6. Numerical simulations were carried out in a similar manner as shown in Fig 8G–8J. Equations and regulatory functions were used as in S1 Table with parameter values of *K* = 2, *A* = *E*_*p*_ = *E*_*q*_ = *G*_*p*_ = *V* = 1.0, *p* = *q* = 10.0, *G*_*a*_ = 0.2, *G*_*x*_ = 0.5, *r* = 2.0, *m* = 6.0, *D*_*a*_ = 0.0, 30.0, 10.0, 1.0, or 0.1 (A–E and J, F, G, H, or I, respectively), and *D*_*x*_ = 30.0, 10.0, 1.0, 0.1, or 0.0 (A, B, C, D, or E–J, respectively).
(TIFF)
