# Supplementary material for: Spatial regularity control of phyllotaxis pattern generated by the mutual interaction between auxin and PIN1
Source: PLoS Comput Biol. 2018 Apr 3;14(4):e1006065. doi: 10.1371/journal.pcbi.1006065 (PMC5882125; doi:10.1371/journal.pcbi.1006065)
Supplement: S1 Text — (PDF) [file pcbi.1006065.s001.pdf]

Spatial regularity control of phyllotaxis pattern generated by the mutual interaction between auxin and PIN1

Hironori Fujita[1,2*], Masayoshi Kawaguchi[1,2]

[1]National Institute for Basic Biology, Okazaki, Aichi, Japan

[2]Department of Basic Biology, School of Life Science, SOKENDAI (The Graduate University for Advanced Studies), Okazaki, Aichi, Japan

[*]hfujita@nibb.ac.jp

S1 Text. Linear stability analysis

(i) Eigenvalue of circulant matrix

Let's consider the following matrix of $N \times N$ elements ($N \geq 6$):

$$\begin{pmatrix} c_0 & c_1 & \cdots & c_{N-2} & c_{N-1} \\ c_{N-1} & c_0 & \cdots & c_{N-3} & c_{N-2} \\ \vdots & \vdots & \ddots & \vdots & \vdots \\ c_2 & c_3 & \cdots & c_0 & c_1 \\ c_1 & c_2 & \cdots & c_{N-1} & c_0 \end{pmatrix} \quad \text{(S1)}$$

where c_n $(n = 0,1,\cdots,N-1)$ are constants. Because this is a circulant matrix, the eigenvalues and their respective eigenvectors can be described respectively by

$$\lambda_k = \sum_{n=0}^{N-1} c_n \, \omega_k^n \quad \text{(S2)}$$

$$\boldsymbol{u}_k \propto (1, \omega_k, \omega_k^2, \cdots, \omega_k^{N-1})^T \quad \text{(S3)}$$

where $\omega_k \equiv e^{i\frac{2\pi k}{N}}$ $(k = 0,1,\cdots,N-1)$. Particularly in the case of $c_1 = c_{N-1}$,

$c_2 = c_{N-2}$, and $c_3 = \cdots = c_{N-3} = 0$, Eq S2 becomes

$$\lambda_k(\nu) = 4c_2\nu^2 + 2c_1\nu + c_0 - 2c_2 \tag{S4}$$

where $\nu \equiv \cos(2\pi k/N) \in [-1,1]$.

(ii) Model O

We considered a one-dimensional cell array with total cell number *N* under the periodic boundary condition, and performed linear stability analysis with reference to Jönsson *et al.* [1]. In this situation ($K = 2$, $j = i \pm 1$, and $i = 1, \cdots, N$), Model O (Eqs 1–3) becomes

$$\frac{da_i}{dt} = G_a(A - a_i) - (f_{i,i+1} + f_{i,i-1}) + D_a(a_{i+1} + a_{i-1} - 2a_i) \tag{S5}$$

$$\frac{dp_{i,j}}{dt} = G_p\left(2p\frac{\varphi_0(a_j)}{\varphi_0(a_{i+1}) + \varphi_0(a_{i-1})} - p_{i,j}\right) \tag{S6}$$

$$f_{i,j} = E_p(p_{i,j}a_i - p_{j,i}a_j) \tag{S7}$$

For simplification, we used the equilibrium values of PIN1 densities by solving $dp_{i,j}/dt = 0$:

$$p_{i,j} = 2p\frac{\varphi_0(a_j)}{\varphi_0(a_{i+1}) + \varphi_0(a_{i-1})} \tag{S8}$$

By substituting Eqs S7 and S8 into Eq S5, we can obtain a system of differential equations with auxin concentrations a_i, which is denoted here by $da_i/dt = f_i(a_1, \cdots, a_N)$. The Jacobian matrix of this dynamics at the equilibrium ($a_1 = \cdots = a_N = a_{eq}$) (Eq 4) is described by

$$\left.\frac{\partial f_i}{\partial a_j}\right|_{eq} = \begin{cases} c_0 \equiv -(G_a + 2c_1 + 2c_2) \quad (|i-j| = 0) \\ c_1 \equiv E_p p + D_a \quad (|i-j| = 1 \text{ or } N-1) \\ c_2 \equiv -\dfrac{E_p p a_{eq} \varphi_0'(a_{eq})}{2\varphi_0(a_{eq})} \quad (|i-j| = 2 \text{ or } N-2) \\ 0 \quad (\text{otherwise}) \end{cases} \quad \text{(S9)}$$

Because this is a circulant matrix, its eigenvalue $\lambda_k(\nu)$ is given by Eq S4 and satisfies

$$\lambda_k(-1) = -(4E_p p + 4D_a + G_a) < 0 \quad \text{(S10)}$$

$$\lambda_k(1) = -G_a < 0 \quad \text{(S11)}$$

Thereby, if $c_2 \geq 0$ then we always have $\lambda_k(\nu) < 0$ for $-1 \leq \nu \leq 1$, indicating that the equilibrium is always stable. In contrast, if $c_2 < 0$, $\lambda_k(\nu) > 0$ for some $\nu \in [-1,1]$ occurs when

$$|\nu_*| < 1 \text{ and } \lambda_k(\nu_*) > 0 \quad \text{(S12)}$$

where $\nu_* \equiv -c_1/4c_2$. $\lambda_k(\nu)$ reaches the maximum at $\nu = \nu_*$, which corresponds to wavenumber $k = k_*$ and wavelength L_*, satisfying

$$L_* = N/k_* = 2\pi/\cos^{-1}(\nu_*) \quad \text{(cells)} \quad \text{(S13)}$$

This indicates that, if Eq S12 is satisfied, the equilibrium becomes spatially unstable and patterns with the wavelength L_* grow fastest. Using Eq S9, ν_* becomes

$$\nu_* = \frac{1+R_a}{2} \frac{\varphi_0(a_{eq})}{a_{eq}\varphi_0'(a_{eq})} \quad \text{(S14)}$$

where $R_a \equiv D_a/E_p p$.

(iii) Model A

We consider a one-dimensional array of alternating cell and apoplast with total cell number *N* under the periodic boundary condition. In this situation ($K = 2$, $j = i \pm 1$, and $i = 1, \cdots, N$), Model A (Eqs 11–14) becomes

$$\frac{da_i}{dt} = G_a(A - a_i) - (f_{i,i+1} + f_{i,i-1}) + D_a(a'_i + a'_{i-1} - 2a_i) \tag{S15}$$

$$\frac{da'_i}{dt} = -G_a a'_i + \frac{1}{V}(f_{i,i+1} + f_{i+1,i}) + \frac{D_a}{V}(a_i + a_{i+1} - 2a'_i) \tag{S16}$$

$$\frac{dp_{i,j}}{dt} = G_p\left(2p\frac{\varphi_a(a'_{i,j})}{\varphi_a(a'_i) + \varphi_a(a'_{i-1})} - p_{i,j}\right) \tag{S17}$$

$$f_{i,j} = E_p p_{i,j} a_i - E_q q a'_{i,j} \tag{S18}$$

where $a'_i \equiv a'_{i,i+1} (= a'_{i+1,i})$. For simplification, we used the analytically calculated equilibrium values of cytosolic auxin concentrations and PIN1 densities (i.e., $da_i/dt = dp_{i,j}/dt = 0$):

$$a_i = \big(G_a A + (E_q q + D_a)(a'_i + a'_{i-1})\big)/\big(E_p(p_{i,i+1} + p_{i,i-1}) + 2D_a + G_a\big) \tag{S19}$$

$$p_{i,j} = 2p\varphi_a(a'_{i,j})/(\varphi_a(a'_i) + \varphi_a(a'_{i-1})) \tag{S20}$$

By substituting Eqs S18–S20 into Eq S16, we can obtain a system of differential equations with apoplast auxin concentrations a'_i, which is described here by $da'_i/dt = f_i(a'_1, \cdots, a'_N)$. The Jacobian matrix of this dynamics at the equilibrium ($a'_1 = \cdots = a'_N = a'_{eq}$) (Eq 15) is given by

$$\left.\frac{\partial f_i}{\partial a'_j}\right|_{eq} = \begin{cases} c_0 \equiv \alpha + \beta - \big(2(E_q q + D_a)/V + G_a\big) & (|i-j| = 0) \\ c_1 \equiv (\alpha - \beta)/2 & (|i-j| = 1 \text{ or } N-1) \\ 0 & (\text{otherwise}) \end{cases} \tag{S21}$$

where $\alpha \equiv 2(E_p p + D_a)(E_q q + D_a)/V(2E_p p + 2D_a + G_a)$ and $\beta \equiv E_p p\big(2(E_q q + D_a)a'_{eq} + G_a A\big)\varphi'_a(a'_{eq})/V(2E_p p + 2D_a + G_a)\varphi_a(a'_{eq})$.

Because this is a circulant matrix, according to Eq S4, its eigenvalues are described by

$$\lambda_k(\nu) = 2c_1\nu + c_0 \tag{S22}$$

which is a linear function of $\nu \equiv \cos(2\pi k/N) \in [-1,1]$ and satisfies

$$\lambda_k(1) = -G_a\left(\frac{2\left(E_q q + D_a\right)}{V\left(2E_p p + 2D_a + G_a\right)} + 1\right) < 0 \tag{S23}$$

Thereby, $\lambda_k(\nu) > 0$ for some $\nu \in [-1,1]$ occurs when

$$\lambda_k(-1) > 0 \tag{S24}$$

If Eq S24 is satisfied, $\lambda_k(\nu)$ becomes positive for $-1 \le \nu < -c_0/2c_1$ and reaches the maximum at $\nu = \nu_* = -1$, indicating that the wavelength of patterns with the highest growth rate is independent of parameter values and always given by

$$L_* \equiv N/k_* = 2 \text{ (apoplast spaces)} \tag{S25}$$

where $\nu_* \equiv \cos(2\pi k_*/N) = -1$.

(iv) Model A with direct apoplast diffusion

The direct apoplast diffusion of auxin can be added to Model A (Eqs S15–18) by replacing Eq S16 with

$$\frac{da_i'}{dt} = -G_a a_i' + \frac{1}{V}(f_{i,i+1} + f_{i+1,i}) + \frac{D_a}{V}(a_i + a_{i+1} - 2a_i') + \frac{D_{a2}}{V}(a_{i+1}' + a_{i-1}' - 2a_i') \tag{S26}$$

where D_{a2} is the diffusion coefficient between neighboring apoplast spaces. Accordingly, Eq S21 is replaced by

$$\left.\frac{\partial f_i}{\partial a_j'}\right|_{eq} = \begin{cases} c_0' \equiv c_0 - 2D_{a2}/V & (|i-j| = 0) \\ c_1' \equiv c_1 + D_{a2}/V & (|i-j| = 1 \text{ or } N-1) \\ 0 & (\text{otherwise}) \end{cases} \tag{S27}$$

where c_0 and c_1 have the same notations as in Eq S21. This Jacobian matrix corresponds to that of Model A with substituting c_0 and c_1 for c_0' and c_1', respectively, indicating that the direct apoplast diffusion has no essential effects on the

spatial regularity control.

(v) Model B6

As with Model A, we consider a one-dimensional array of alternating cell and apoplast with total cell number *N* under the periodic boundary condition. In the absence of the regulation of apoplast auxin on PIN1 polarization ($K = 2,\ n = 0,\ j = i \pm 1$, and $i = 1, \cdots, N$), Model B6 (Eqs 11–13, 18, 19, and 26) becomes

$$\frac{da_i}{dt} = G_a(A - a_i) - (f_{i,i+1} + f_{i,i-1}) + D_a(a'_i + a'_{i-1} - 2a_i) \tag{S28}$$

$$\frac{da'_i}{dt} = -G_a a'_i + \frac{1}{V}(f_{i,i+1} + f_{i+1,i}) + \frac{D_a}{V}(a_i + a_{i+1} - 2a'_i) \tag{S29}$$

$$\frac{dp_{i,j}}{dt} = G_p\left(2p\frac{\varphi_x\left(x'_{i,j}\right)}{\varphi_x(x'_i) + \varphi_x(x'_{i-1})} - p_{i,j}\right) \tag{S30}$$

$$\frac{dx_i}{dt} = G_x(\theta(a_i) - x_i) + D_x(x'_i + x'_{i-1} - 2x_i) \tag{S31}$$

$$\frac{dx'_i}{dt} = -G_x x'_i + \frac{D_x}{V}(x_i + x_{i+1} - 2x'_i) \tag{S32}$$

$$f_{i,j} = E_p p_{i,j} a_i - E_q q a'_{i,j} \tag{S33}$$

where $a'_i \equiv a'_{i,i+1}(= a'_{i+1,i})$ and $x'_i \equiv x'_{i,i+1}(= x'_{i+1,i})$. For simplification, we used the analytically calculated equilibrium values of apoplast auxin concentrations, PIN1 densities, and *X* concentrations (i.e., $da'_i/dt = dp_{i,j}/dt = dx_i/dt = dx'_i/dt = 0$):

$$a'_i = \frac{(E_p p_{i,i+1} + D_a)a_i + (E_p p_{i+1,i} + D_a)a_{i+1}}{2E_q q + 2D_a + VG_a} \tag{S34}$$

$$p_{i,j} = 2p\frac{\varphi_x\left(x'_{i,j}\right)}{\varphi_x(x'_i) + \varphi_x(x'_{i-1})} \tag{S35}$$

$$x_i = \frac{G_x\theta(a_i) + D_x(x'_i + x'_{i-1})}{2D_x + G_x} \approx \frac{G_x\theta(a_i) + 2D_x x'_{eq}}{2D_x + G_x} \tag{S36}$$

$$x_i' = \frac{D_x(x_i + x_{i+1})}{2D_x + VG_x} \tag{S37}$$

where cytosolic $X\,(x_i)$ is approximated by replacing x_i' with its equilibrium x_{eq}' in Eq S36. By substituting Eqs S33–S37 into Eq S28, we can obtain a system of differential equations with cytosolic auxin concentrations a_i, which is denoted here by $da_i/dt \approx f_i(a_1, \cdots, a_N)$. The Jacobian matrix of this dynamics at the equilibrium ($a_1 = \cdots = a_N = a_{eq}$) (Eq 15) is approximately given by

$$\left.\frac{\partial f_i}{\partial a_j}\right|_{eq} \approx \begin{cases} c_0 \equiv 2c_1 - 2c_2 - \left(2E_p p + 2D_a + G_a\right) & (|i-j| = 0) \\ c_1 \equiv \gamma\left(E_p p + D_a\right) & (|i-j| = 1 \text{ or } N-1) \\ c_2 \equiv -\dfrac{\gamma\kappa E_p p}{2}\dfrac{a_{eq}\theta'(a_{eq})\varphi_x'\left(x_{eq}'\right)}{\varphi_x\left(x_{eq}'\right)} & (|i-j| = 2 \text{ or } N-2) \\ 0 & (\text{otherwise}) \end{cases} \tag{S38}$$

where $\gamma \equiv (E_q q + D_a)/(2E_q q + 2D_a + VG_a)$ and $\kappa \equiv D_x G_x/(2D_x + G_x)(2D_x + VG_x)$. Because this is a circulant matrix, its eigenvalue $\lambda_k(\nu)$ is given by a quadratic function of $\nu \in [-1,1]$ (Eq S4) and satisfies

$$\lambda_k(-1) \approx -\left(2E_p p + 2D_a + G_a\right) < 0 \tag{S39}$$

$$\lambda_k(1) \approx -G_a - \frac{2\left(E_p p + D_a\right)VG_a}{2E_q q + 2D_a + VG_a} < 0 \tag{S40}$$

Therefore, by a similar argument to that of Model O, we can obtain the following approximate equations corresponding to Eqs S4, S12, and S13:

$$\lambda_k(\nu) \approx 4c_2\nu^2 + 2c_1\nu + c_0 - 2c_2 \tag{S41}$$

$$|\nu_*| < 1 \text{ and } \lambda_k(\nu_*) > 0 \tag{S42}$$

$$L_* \approx 2\pi/\cos^{-1}(\nu_*) \quad (\text{cells}) \tag{S43}$$

where $\nu \equiv \cos(2\pi k/N) \in [-1,1]$ and $\nu_* \approx -c_1/4c_2$. Using Eq S38, ν_* becomes

$$\nu_* \approx \frac{(1+R_a)(1+2R_x)(V+2R_x)}{2R_x}\frac{\varphi_x(x'_{eq})}{a_{eq}\theta'(a_{eq})\varphi'_x(x'_{eq})} \tag{S44}$$

where $R_a \equiv D_a/E_p p$ and $R_x \equiv D_x/G_x$. In the case of regulatory functions $\theta(a_i) = 2{a_i}^r/({a_{eq}}^r + {a_i}^r)$ and $\varphi_x(x'_{i,j}) = {x'_{i,j}}^m$, Eq S44 becomes

$$\nu_* \approx \frac{2(1+R_a)}{rm}\frac{(1+2R_x)(V+2R_x)}{V+2(1+V)R_x} \tag{S46}$$

Reference

1. Jönsson H, Heisler MG, Shapiro BE, Meyerowitz EM, Mjolsness E. An auxin-driven polarized transport model for phyllotaxis. Proc Natl Acad Sci USA. 2006;103(5):1633-8. doi: 0509839103 [pii] 10.1073/pnas.0509839103. PubMed PMID: 16415160; PubMed Central PMCID: PMCPMC1326488.
