# Supplementary material for: Spatial regularity control of phyllotaxis pattern generated by the mutual interaction between auxin and PIN1
Source: PLoS Comput Biol. 2018 Apr 3;14(4):e1006065. doi: 10.1371/journal.pcbi.1006065 (PMC5882125; doi:10.1371/journal.pcbi.1006065)
Supplement: S1 Table — (PDF) [file pcbi.1006065.s002.pdf]

S1 Table. Summary of equations and regulatory functions used in numerical simulations

Diffusion type	Model	Figure	Equation	Regulatory function
	Model O	Fig 2	Eqs 1–3 ($K = 2$)	$\varphi_0(a_j) = {a_j}^n$
	Model O	Fig 3A	Eqs 1–3 ($K = 6$)	$\varphi_0(a_j) = \dfrac{{a_j}^n}{(Ra_{eq})^n + {a_j}^n}$
Simple diffusion of auxin	Model A	Fig 4	Eqs 11–14 ($K = 2$)	$\varphi_a(a'_{i,j}) = {a'_{i,j}}^n$
Simple and symplast diffusions of auxin	Model A	Fig 5A and 5B	Eqs 12–14 and 16 ($K = 2$)	
Simple and apoplast diffusions of auxin	Model A	Fig 5C and 5D	Eqs 11, 13, 14, and 17 ($K = 2$)	
Simple diffusion of auxin	Model A	Fig 3B	Eqs 11–14 ($K = 6$)	$\varphi_a(a'_{i,j}) = \dfrac{{a'_{i,j}}^n}{(Ra'_{eq})^n + {a'_{i,j}}^n}$
Simple diffusion of molecule X	Model B1	Fig 6A and 6B	Eqs 11, 12, 14, 18, 19, and 21 ($K = 2$)	$\theta(a_i) = \dfrac{2{a_i}^r}{{a_{eq}}^r + {a_i}^r}$ $\varphi_a(a'_{i,j}) = {a'_{i,j}}^n$ $\psi_1(x_i) = \dfrac{2{x_i}^m}{{x_{eq}}^m + {x_i}^m}$
	Model B2	Fig 6C and 6D	Eqs 11–13, 18, 19, and 22 ($K = 2$)	
		S1 Fig	Eqs 11–13, 18, 19, and 22 ($K = 6$)	
	Model B3	Fig 6E and 6F	Eqs 12–14, 18, 19, and 23 ($K = 2$)	
	Model B4	Fig 6G and 6H	Eqs 11, 12, 14, 18, 19, and 24 ($K = 2$)	$\theta(a_i) = \dfrac{2{a_i}^r}{{a_{eq}}^r + {a_i}^r}$ $\varphi_a(a'_{i,j}) = {a'_{i,j}}^n$ $\psi_2(x'_{i,j}) = \dfrac{2{x'_{i,j}}^m}{{x'_{eq}}^m + {x'_{i,j}}^m}$
	Model B5	Fig 6I and 6J	Eqs 11, 12, 14, 18, 19, and 25 ($K = 2$)	
		Fig S2	Eqs 11, 12, 14, 18, 19, and 25 ($K = 6$)	

	Model B6	Fig 6K and 6L	Eqs 11–13, 18, 19, and 26 ($K = 2$)	$\theta(a_i) = \frac{2{a_i}^r}{{a_{eq}}^r + {a_i}^r}$<br>$\varphi_a(a'_{i,j}) = {a'_{i,j}}^n$<br>$\varphi_x(x'_{i,j}) = {x'_{i,j}}^m$
		Fig 8 and S4 Fig	Eqs 11–13, 18, 19, and 26 ($K = 2$)	$\theta(a_i) = \frac{2{a_i}^r}{{a_{eq}}^r + {a_i}^r}$<br>$\varphi_a(a'_{i,j}) = 1$<br>$\varphi_x(x'_{i,j}) = {x'_{i,j}}^m$
		Fig 3C	Eqs 11–13, 18, 19, and 26 ($K = 6$)	$\theta(a_i) = a_i$<br>$\varphi_a(a'_{i,j}) = 1$<br>$\varphi_x(x'_{i,j}) = \frac{{x'_{i,j}}^m}{(Rx'_{eq})^m + {x'_{i,j}}^m}$
Symplast diffusion of molecule X	Model B1	S3A and S3B Fig	Eqs 11, 12, 14, 21, 27, and 28 ($K = 2$)	$\theta(a_i) = \frac{2{a_i}^r}{{a_{eq}}^r + {a_i}^r}$<br>$\varphi_a(a'_{i,j}) = {a'_{i,j}}^n$<br>$\psi_1(x_i) = \frac{2{x_i}^m}{{x_{eq}}^m + {x_i}^m}$
	Model B2	S3C and S3D Fig	Eqs 11–13, 22, 27, and 28 ($K = 2$)	
	Model B3	S3E and S3F Fig	Eqs 12–14, 23, 27, and 28 ($K = 2$)	
Apoplast diffusion of molecule X	Model B4	S3G and S3H Fig	Eqs 11, 12, 14, 24, 30, and 31 ($K = 2$)	$\theta(a_i) = \frac{2{a_i}^r}{{a_{eq}}^r + {a_i}^r}$<br>$\varphi_a(a'_{i,j}) = {a'_{i,j}}^n$<br>$\psi_2(x'_{i,j}) = \frac{2{x'_{i,j}}^m}{{x'_{eq}}^m + {x'_{i,j}}^m}$
	Model B5	S3I and S3J Fig	Eqs 11, 12, 14, 25, 30, and 31 ($K = 2$)	
	Model B6	S3K and S3L Fig	Eqs 11–13, 26, 30, and 31 ($K = 2$)	$\theta(a_i) = \frac{2{a_i}^r}{{a_{eq}}^r + {a_i}^r}$<br>$\varphi_a(a'_{i,j}) = {a'_{i,j}}^n$<br>$\varphi_x(x'_{i,j}) = {x'_{i,j}}^m$
