# Supplementary figures and images for: Spatial regularity control of phyllotaxis pattern generated by the mutual interaction between auxin and PIN1
Source: PLoS Comput Biol. 2018 Apr 3;14(4):e1006065. doi: 10.1371/journal.pcbi.1006065 (PMC5882125; doi:10.1371/journal.pcbi.1006065)

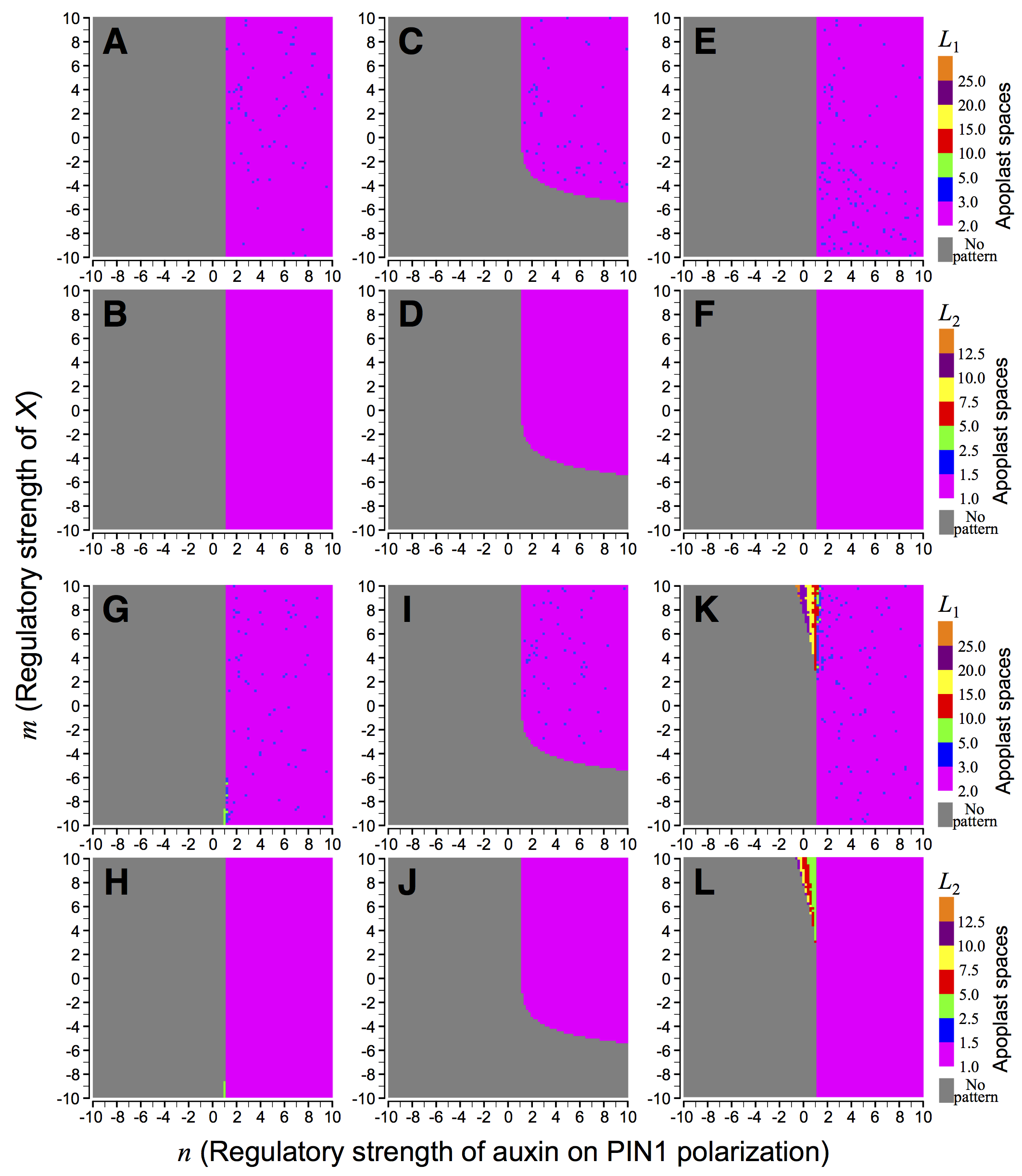

Supplement: S3 Fig — Wavelength of auxin maxima pattern (L1) (A, C, E, G, I, and K) and average size of auxin maximum (L2) (B, D, F, H, J, and L) were determined in Model B1 (A and B), Model B2 (C and D), Model B3 (E and F), Model B4 (G and H), Model B5 (I and J), and Model B6 (K and L). The symplast diffusion (A–F) or apoplast diffusion (G–L) of molecule X was used instead of the simple diffusion between cytoplasm and apoplast in Fig 5 (Fig 1F). Numerical simulations were carried out in a similar manner as shown in Fig 6. Equations and regulatory functions are used as in S1 Table with parameter values of K = 2, A = Ep = Eq = Gx = Gp =Da = V = r = 1.0, p = q = 10.0, and Ga = 0.2 (A–L), Dx1 = 10.0 (A–F), Dx2 = 10.0 (G–L), and S = 1.0 (G–L). (TIFF) [file pcbi.1006065.s005.tiff]

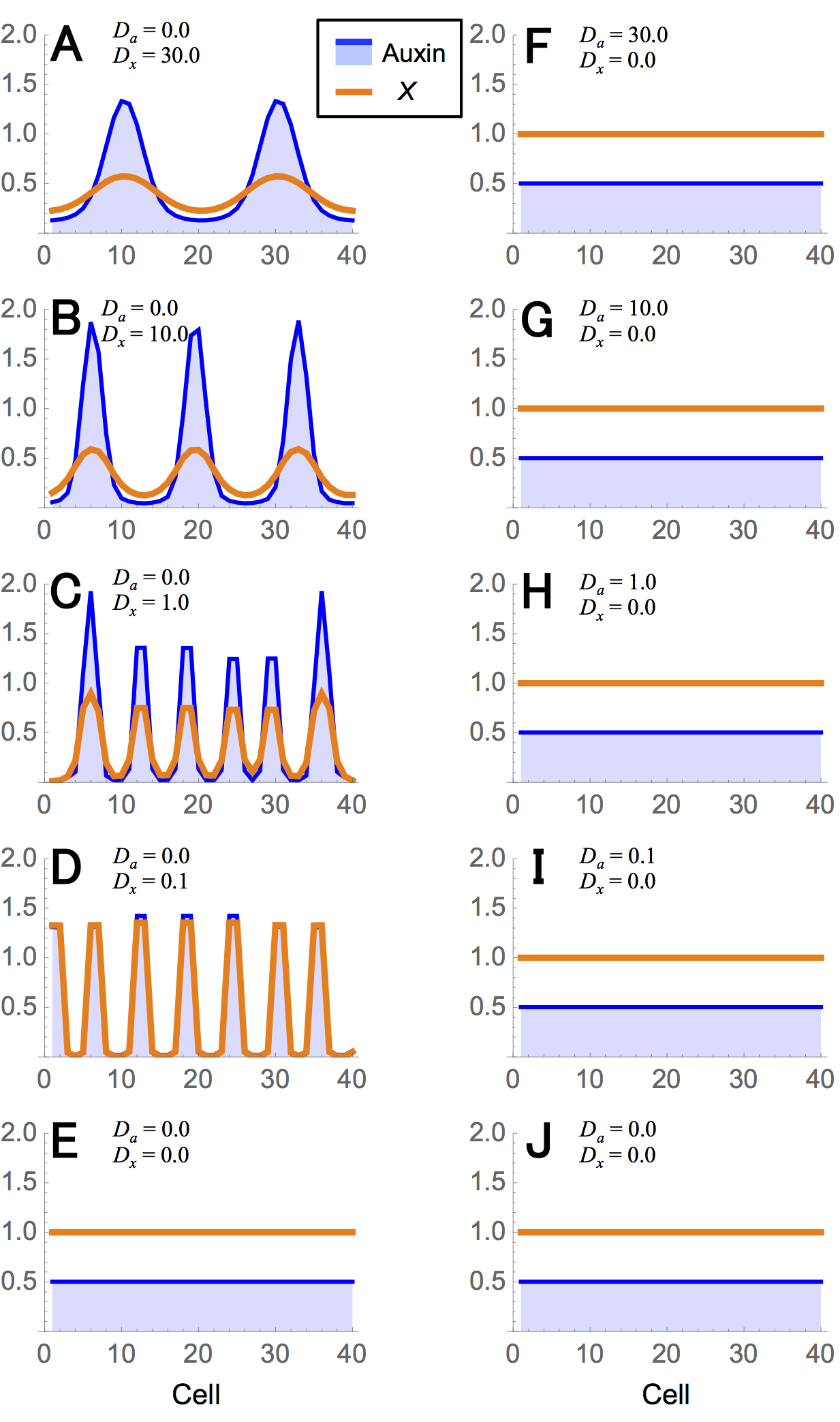

Supplement: S4 Fig — Examples of auxin distribution in the absence of auxin diffusion (Da = 0.0; A–E) or X diffusion (Dx = 0.0; F–J) in Model B6. Numerical simulations were carried out in a similar manner as shown in Fig 8G–8J. Equations and regulatory functions were used as in S1 Table with parameter values of K = 2, A = Ep = Eq = Gp = V = 1.0, p = q = 10.0, Ga = 0.2, Gx = 0.5, r = 2.0, m = 6.0, Da = 0.0, 30.0, 10.0, 1.0, or 0.1 (A–E and J, F, G, H, or I, respectively), and Dx = 30.0, 10.0, 1.0, 0.1, or 0.0 (A, B, C, D, or E–J, respectively). (TIFF) [file pcbi.1006065.s006.tiff]
